# The function and evolution of a genetic switch controlling sexually dimorphic eye differentiation in honeybees

**DOI:** 10.1038/s41467-023-36153-4

**Published:** 2023-01-28

**Authors:** Oksana Netschitailo, Yidong Wang, Anna Wagner, Vivien Sommer, Eveline C. Verhulst, Martin Beye

**Affiliations:** 1 Institute of Evolutionary Genetics, Heinrich-Heine University, Duesseldorf, Germany; 2 Laboratory of Entomology, Wageningen University, Wageningen, the Netherlands

**Keywords:** Gene regulation, Development, Evolutionary developmental biology, Entomology, Molecular evolution

## Abstract

Animals develop sex-specific morphological structures that are diverse between organisms. However, understanding the developmental and evolutionary mechanisms governing these traits is still limited and largely restricted to DM domain genes, which are conserved, sex-specific developmental regulators identified in genetic models. Here, we report a sex-specific developmental regulator gene, *glubschauge* (*glu*) that selectively regulates sexually dimorphic eye differentiation in honeybees. We found that the sex determination gene *feminizer* (*fem*) controls sex-specific splicing of *glu* transcripts, establishing a genetic switch in which Glu proteins with a zinc finger (ZnF) domain are only expressed in females. We showed that female coding sequence was essential and sufficient for partial feminization. Comparative sequence and functional studies revealed that the evolutionary origination of the genetic switch was followed by the mutational origin of the essential ZnF domain. Our results demonstrate that *glu* is a newly evolved sex-specific genetic switch for region-specific regulation of a dimorphic character.

## Abstract (web summary)

Sexual dimorphism results in widely diverse animal forms, but sexual determination is generally attributed to a single gene in animal models. Here they find that the *glu* gene regulates sexual dimorphism of honeybee eyes, demonstrating diversification of genetic programs for dimorphism.

## Introduction

Morphological differences between males and females are very common in animal organisms. The development of such sexual dimorphism directly or indirectly enhances the fitness of the bearer. The exaggerated horns of some beetles and the color patterning and size of the peacock’s tail provide intriguing examples of such sexually dimorphic structures. The evolutionary emergence of new sexual characteristics in different animal lineages establishes remarkable differences among organisms. However, our knowledge about the molecular developmental and evolutionary mechanisms governing sexual dimorphism is still limited. This is partly because the work has been focused on a limited number of sexually dimorphic traits, and systematic searches for developmental regulators have been performed in very few genetic models.

Sex determination establishes a binary signal that is usually transduced via a cascade of genes to sex-specifically controlled developmental regulators responsible for mediating aspects of sexual differentiation^1–3^. In vertebrates, the sex determination pathway determines the sex of the gonad. The gonads produce sex hormones that regulate the sexual fate of the non-gonadal tissues. In invertebrates, the primary sex determination signal is transduced to developmental regulators that are transcription factors. The result is a purely cell autonomous but coordinated decision about the sexual fate. One key developmental regulator for sexual dimorphism in invertebrates is the DM domain gene^1,2,4^. This gene encodes a transcription factor of the DM domain type with an intertwined zinc finger motif-type DNA binding domain and is involved in the specification of reproductive organs^1,5^. Sexual dimorphisms regulated by DM domain genes in other body parts are male tale morphogenesis in *Caenorhabditis elegans*^6,7^, the male-specific antenna and copulatory thoracic hook in the crustacean *Daphnia magna*^8^, sex combs on the male forelegs in *Drosophila melanogaster*^9,10^, sexually dimorphic exaggerated horn structures in some beetles^11–13^ and other characteristics^4,14–18^. A general feature that emerges from these studies is that DM domain genes are developmental regulators that operate as sex-specific genetic switches, since they provide activity either limited to one sex (ON or OFF activity states), or distinct between the sexes. Systematic screens revealed that the *doublesex* (*dsx*) gene (which is one of the DM domain genes in insects) is the main developmental regulator of sexual differentiation in *D. melanogaster*, since it specifies nearly all sexually dimorphic characteristics in this species^19,20^. However, how other sexually dimorphic characteristics are regulated is largely unknown.

Our previous work suggested that besides the reproductive organs the external sexually dimorphic characteristics of the honeybee *Apis mellifera* are not specified by the *dsx* gene^21^. These characteristics include the compound eyes that are approximately fourfold larger in males than in females (see wild type (wt) phenotypes in Supplementary Fig. 1A), which is likely an adaptation to spot males and queens during their mating flight^22–24^. Quantitative measures of eye sizes in *dsx* loss of function mutants showed that the *dsx* gene is not required for the development of the sexually dimorphic eye (Supplementary Fig. 1B).

This knowledge motivated us to systematically search for another sex-specific developmental regulator. We wanted to test the sexually dimorphic function and to examine the evolutionary origin of this other regulator with the aim of deeply understanding the developmental and evolutionary mechanism underlying sexually dimorphic trait formation. We identified a gene, which selectively specifies sexually dimorphic eye differentiation and named it *glubschauge* (*glu*). The gene operates as a sex-specific genetic switch as it provides distinct activities in females and males. Only the female-specific transcripts encode a protein with a zinc finger (ZnF). We further showed that the female-specific coding sequence is essential and sufficient for the partial feminization of the entire structure. Comparative evolutionary sequence and functional studies revealed that the gene was newly recruited to the sex determination pathway, which was followed by the evolutionary gain of the essential ZnF motif. Our results show that *glu* is a newly evolved sex-specific developmental regulator that controls sexual dimorphism of a single structure, the compound eye. Together, these findings suggest a region-specific control in which sexually dimorphic characteristics in different body parts are instructed by different sex-specific developmental regulators, *glu* and *dsx*.

## Results

### *glu* transcripts encode a zinc finger motif only in females

**Fig. 1:**
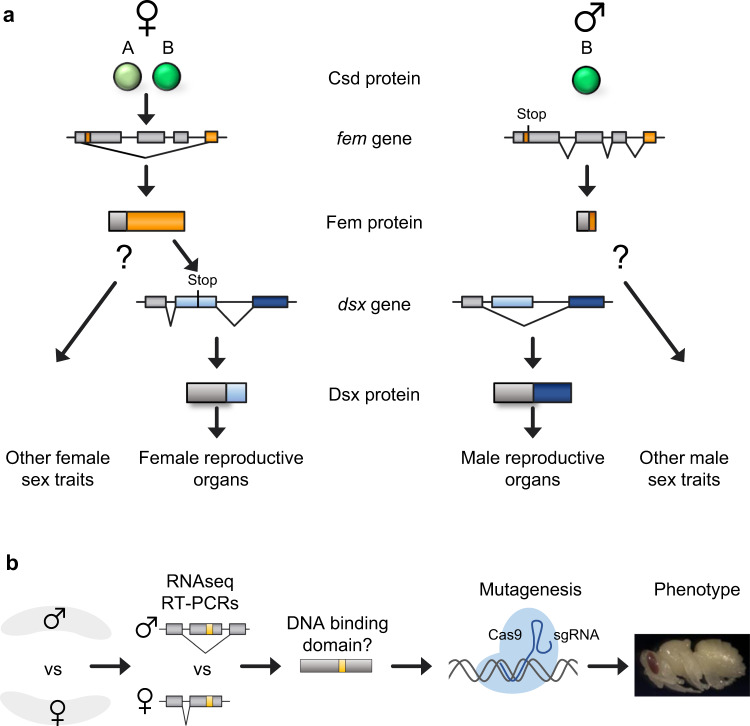
Sex determination in honeybees and the search for another sex-specific developmental regulator. **a** A model of the sex determination pathway with the *dsx* gene as a sex-specific developmental regulator of reproductive organ differentiation. The proteins, as well as the sex-specific splicing of the transcripts are schematically presented. A and B are protein variants derived from different *csd* alleles. Different colors indicate exons and proteins that are sex-specifically spliced or expressed. *csd*: *complementary sex determiner* gene. *fem*: *feminizer* gene. **b** The experimental strategy employed to identify other developmental regulators that are sex-specific spliced and regulated by the *fem* gene. Transcriptome analysis in embryos identified sex-specifically spliced transcripts. The coding sequence analysis identified possible DNA binding domains.

To identify a regulator of sexually dimorphic development other than the *dsx* gene, we screened for sex-specifically spliced transcripts. In honeybees, the heterozygous or homozygous/hemizygous genotype of the *complementary sex determiner* (*csd*) gene is the primary signal of sex determination (Fig. 1a). The *csd* gene regulates female- and male-specific splicing of the *feminizer* (*fem*) transcript generating a functional Fem protein only in females^25,26^. The *fem* gene thereby controls the entire development of either females or males^25,26^. The expressed Fem protein in females directs at least the female-specific splicing of the transcripts of the developmental regulator gene, *dsx*, which specifies gonad development (Fig. 1a)^21^. The male splice variant of the *fem* transcript is produced by default^25,26^. We performed female and male embryonic transcriptome sequencing^27^ and searched for sex-specific splice junctions that are only present in females but not in males (Fig. 1b). These transcripts are possibly regulated by the Fem protein, which is an ortholog of the Transformer protein in *Drosophila*^25,26^. We found that the transcripts of the *glubschauge* gene (*glu*, Gene ID 552468) are sex-specifically spliced, so that proteins with a zinc finger motif can only be expressed in females.

**Fig. 2:**
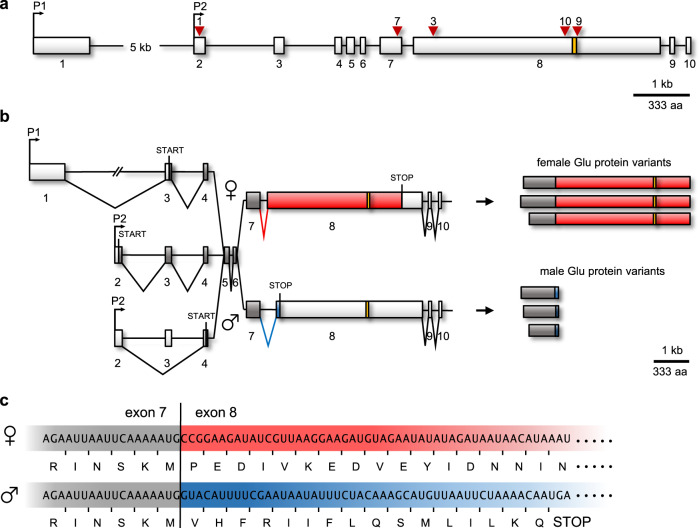
Genomic organization, sex-specific splicing and expression of the *glubschauge* (*glu)* gene. **a** Scheme of the genomic organization of the *glu* gene. The red arrows and the numbers above the genome structure indicate the target sites of the sgRNAs used for CRISPR/Cas9 mutagenesis. **b** Female- and male-specific transcripts and proteins. Boxes denote the exons. Female-specific parts of the ORF are shown in red, male-specific parts are displayed in blue, and common parts are shown in dark gray. The position of the CCHH zinc finger motif (ZnF) is indicated in yellow. **c** The exons 7 and 8 boundary, which is sex-specifically spliced. The coding nucleotide sequence of the mRNA and the encoded amino acids are shown.

We mapped RNA-seq reads and RT–PCR amplicon sequences from male and female embryos and adults to the genomic sequence and found that the *glu* gene consists of 10 exons (Fig. 2a). The transcripts are transcribed from two transcriptional start sites (Fig. 2a, b) and are alternatively spliced, which suggests that at least three possible translation start sites in exons 2, 3 and 4 are used. Splice acceptor sites in exon 8 are sex-specific, which lead to a shift in the open reading frame of exon 8 (ORF; Fig. 2c). Thus the transcripts can express female- and male-specific protein variants, which have shared N-terminal and sex-specific C-terminal amino acid sequences. The shared region is 210 to 316 amino acids long. The female-specific part of the protein consists of 1256 amino acids and harbors a ZnF motif of the CCHH type, representing a possible DNA binding domain^28^. The male-specific region is just 16 amino acid long. We conclude that the *glu* gene can express proteins with a ZnF domain specifically in females, which is absent in males. No conserved motifs other than the ZnF domain were predicted in Glu proteins.

**Fig. 3:**
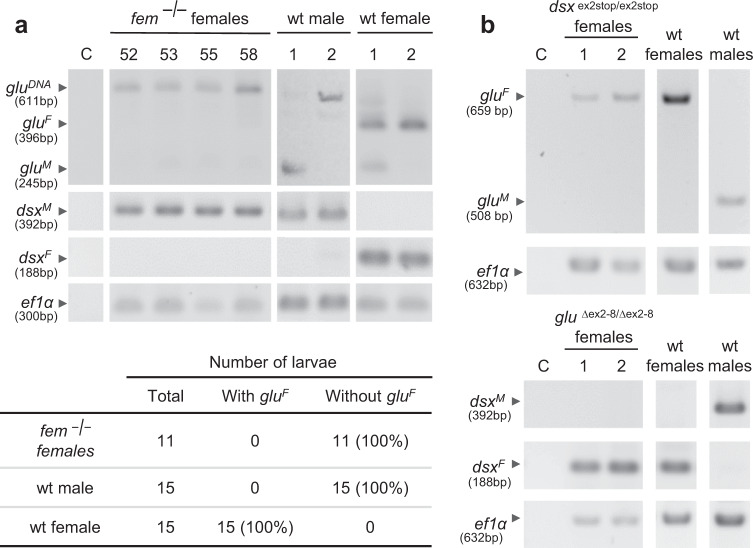
The sex-specific splicing of *glu* is controlled by the *fem* gene. **a** Sex-specific splicing of *glu* transcripts in response to *fem* ^*−*/*−*^ mutations. Individual stage 1 female larvae were analyzed. Upper panel: Size-resolved amplicons from the RT-PCRs, which were semiquantitatively adjusted across individuals using *ef-1α (ef1α, elongation factor 1α*) transcripts as a reference. Female- (*glu*^*F*^) and male-specific (*glu*^*M*^) *glu* fragments were amplified with a single primer pair. Table: The number of individuals in which *glu*^*F*^ was examined. *glu*^*M*^ transcripts showed low abundance and were inconsistently amplified in the mutants. *glu*^*DNA*^: DNA. **b** The *glu* splicing in *dsx* ^*stop/stop*^ female mutants and *dsx* splicing in *glu* ^∆ex2-8/∆ex2-8^ female mutants. The antennae from 4 to 5 pupae or adults were pooled and analyzed. wt: wild type controls. C: negative control for PCR.

To determine whether the *glu* gene is controlled by the *fem* gene, we introduced stop codons into the *fem* gene and studied the sexual splicing of *glu* transcripts. Early stop codons were introduced via frame-shift mutations in exon 3 and both alleles (Supplementary Fig. 2) using the CRISPR/Cas9 method^29^ and the efficient somatic mutation approach^21^. These *fem* ^*−*/*−*^ mutations mimic the male regulatory state (Fig. 1a)^26^. We screened for mutant individuals with no mosaicism^21^, which we identified by the deep sequencing of the amplicons for each individual (Supplementary Table 1). We confirmed that the *fem* ^*−*/*−*^ mutation was a loss-of-function mutation by detecting only the male *dsx* transcript in genetic females (Fig. 3a and Supplementary Table 2). In these *fem* ^*−*/*−*^ females, only the male *glu* splice variant was detected, while in wild type females, only the female *glu* transcript was found (Fig. 3a). This shift in splicing demonstrates that the female splicing of *glu* transcripts is directly or indirectly controlled by the *fem* gene.

To further understand whether *glu* and *dsx* are parallel operating genes under control of the *fem* gene, we studied *glu* splicing in *dsx* loss of function mutants and *dsx* splicing in *glu* loss of function mutants. Again, biallelic mutations were generated for the *dsx* gene in females (*dsx* ^*stop/stop*^)^21^ and the *glu* gene (*glu* ^∆ex2-8/∆ex2-8^; sgRNAs 1 and 3) using CRSPR/Cas9 method. We observed that the lack of *dsx* activity is not affecting *glu* splicing (Fig. 3b). Further, the lack of *glu* activity does not influence *dsx* splicing (Fig. 3b). These results showed that *glu* transcript splicing does not depend on *dsx* activity and that *dsx* transcript splicing does not require *glu* activity. We conclude that *dsx* and *glu* are two sex-specific controlled genes, which operate in parallel branches of the sex determining pathway under control of the *fem* gene.

**Fig. 4:**
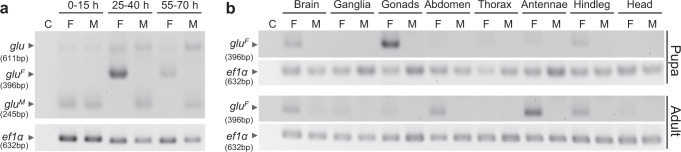
The onset of sex-specific splicing in embryos and the tissue-specific expression of *glu* transcripts. **a** Sex-specific *glu* transcripts at different embryonic stages: cellularization and onset of blastoderm (0–15 h after egg laying), end of blastoderm and gastrulation stage (25–40 h), and larval completion (55–70 h)^69^. Semiquantitative RT-PCR was performed on pools of embryos. **b** Tissue-specific expression of *glu*^*F*^ in males and females at pupal stage 4 and in 10-day-old male and female adults. Semiquantitative RT-PCR results were adjusted across samples using *ef1α* transcript levels as a reference. The abdomen sample does not contain gonads and ganglia. Three biological replicates were conducted. *glu*^*M*^ transcripts in pupae and adults were not amplified (Supplementary Fig. 4). F: female; M: male; *ef1α*: *elongation factor 1α*; C: negative control for PCR.

To determine whether the male transcript is the default regulatory state, which does not rely on input from the *fem* gene, we studied *glu* splicing at different embryonic stages before and after the onset of the sex determination pathway^26,30^. In 0- to 15-hour-old embryos, we detected only one variant that is male-specific at later stages (Fig. 4a). From 25 h onward, with the onset of the sex determination pathway and the alternative splicing of *fem* transcripts after 24–39 h at the cellular blastoderm stage^26,30^, the *glu* gene showed female-specific splicing. The male-specific transcript is the default regulatory state that switches to the female-determined state via Fem protein-mediated splicing. Collectively, these results establish that the *glu* gene operates as a genetic switch with two activity states via sex-specific splicing of transcripts. These transcripts can express different protein activities in females and males.

Next, we examined whether the *glu* gene was tissue-specifically transcribed, which would indicate region-specific expression of the *glu* gene controlled by the developmental programming of general body patterning. At the red eye pupal stage (stage 4), we reliably detected *glu*^*F*^ (female) transcripts in the brain (including the tissues of the complex eyes), gonads and hind legs among all three replicates (Fig. 4b) but not in the other examined tissues. In adult honeybee females, we found that *glu*^*F*^ was expressed in the brain, abdomen, antenna and hind legs (Fig. 4b). We never detected *glu*^*F*^ expression in the thorax or head capsule of adult bees. Intriguingly, we were never able to reliably amplify the male variant in pupae and adults (Supplementary Fig. 3), suggesting an absence of male transcripts. We conclude that the male-specific transcript represents a non-active state of the genetic switch. Since, the male *fem* transcripts are also lacking in pupae^26^, it is possible that the early stop codons induce nonsense-mediated decay of the male-specific *glu*^*M*^ and *fem*^*M*^ transcripts^31^. Collectively, these results show that the *glu* gene is a region-´and sex-specific genetic switch, which can provide an activity limited to only one sex and this in specific tissues.

### The *glu* gene is a regulator of sex-specific eye morphology

**Fig. 5:**
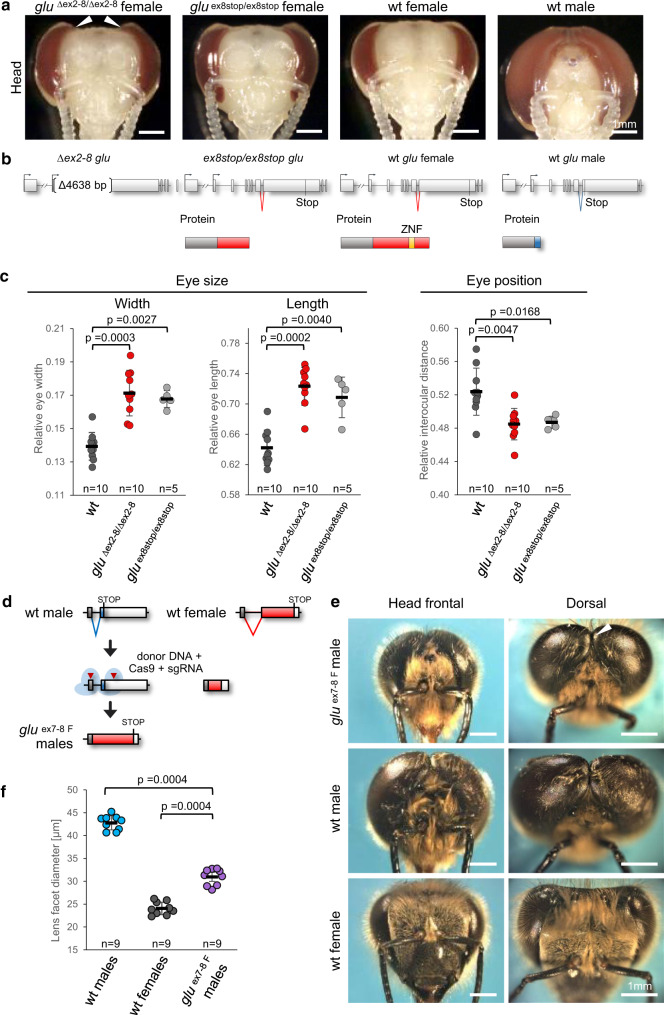
The *glu* gene is essential for female-specific eye formation and sufficient for a feminization of male eye morphology. **a** Head morphology of female *glu* ^∆ex2-8/∆ex2-8^ (*n* = 10) and *glu* ^*ex8stop/ ex8stop*^ (*n* = 5) mutants at pupal stage 4 (red eye stage). Arrow heads mark the male-like dorsal extension of the compound eye. **b** The introduced DNA mutations and the expected protein products are schematically presented. **c** Relative eye width: eye width relative to head width. Relative eye length: eye length relative to head length. Relative interocular distance: interocular distance relative to head width (two-tailed Mann–Whitney U test). Means and standard deviations are shown. **d** Schematic presentation of generating *glu* ^*ex7-8 F*^ mutation in males. **e** Head morphology of feminized *glu* ^*ex7-8 F*^ males (*n* = 9) at the adult stage. Arrow head marks a reduction of dorsal closure in mutant males. **f** Dorsal lens facet diameter, two-tailed Mann–Whitney U test. Means and standard deviations are shown. wt: wild type.

To understand whether the sex-specifically spliced *glu* gene is a developmental regulator of sexual dimorphism, we mutated *glu* in female embryos using the CRISPR/Cas9 method, reared larvae to the pupal stage with worker nutrition and screened for non-mosaic individuals in which both alleles were mutated using deep sequencing of amplicons^21,29^. Genetic females homozygous for exon 2 to exon 8 deletions (*glu* ^*∆ex2-8/∆ex2-8*^, sgRNA1 and 3) developed larger eyes with a male-like dorsal extension (arrow heads, Fig. 5a). Their relative compound eye length and width were significantly greater, while the relative interocular distance of their compound eyes was significantly shorter relative to wild type females (Fig. 5c). These results indicate a partial loss of female characteristics and partial gain of male characteristics. Other sexually dimorphic characteristics of external body morphology and reproductive organs (to the level of stereomicroscope detection) were the same to those of the wild type females (Supplementary Fig. 4) suggesting that the gross developmental function of *glu*^*F*^ is restricted to the region of the compound eye. To understand whether the *glu* gene regulates female-specific and not general developmental characteristics, we compromised specifically the female limited part of the protein. We introduced stop codons in exon 8 using sgRNA 10 so that the female-specific CCHH ZnF motif together with the last 254 to 291 amino acids were not expressed. The *glu* ^*ex8stop/ex8stop*^ females showed the same phenotype as the *glu* ^*∆ex2-8/∆ex2-8*^ females (they were not significantly different) while we again found the effects on relative eye width, length and interocular distance (Fig. 5a–c). This result demonstrates that *glu*^*F*^ transcripts encode a developmental regulator of sexually dimorphic eye development. Next, we wanted to examine whether *glu*^*M*^ transcript is required for male characteristics, despite the lack of expression in male pupae and adults. However, we were not able to test this hypothesis, since already the rearing of pupal control males failed (despite a similar number of eggs) suggesting that our rearing procedure cannot be easily applied to males.

Having shown that the female-specific coding sequence is essential, we next asked whether it is sufficient to feminize the entire structure of the compound eye. We deleted the intron 7 sequence and fused the exon 7/8 sequences using CRISPR/Cas9-mediated homology-directed repair so that the *glu* transcripts present in males encoded only female-specific Glu proteins (Fig. 5d, sgRNAs 3 and 7). The eyes of these *glu* ^*ex7-8 F*^ (haploid) males were smaller in the frontal view (Fig. 5e), while the dorsal closure of the eyes was less pronounced (arrow heads, Fig. 5e), indicating a partial shift from male to female eye morphology that affects the entire eye structure. Next, we asked whether the dorsal lens facets of the compound eyes were feminized. The sizes of the dorsal lens facets in honeybees are extremely sexually dimorphic and contribute to the overall sex-specific size difference of the compound eye^23,24^. The larger male facets show higher light sensitivity and represent an adaptation to spot drones and queens during the male’s mating flight. We found that the dorsal lens facets of the *glu* ^*ex7-8 F*^ males were substantially feminized (*p* < 0.001, Fig. 5f) and nearly showed an entire shift in their size compared to wild type females. These results suggest that the *glu* ^*ex7-8 F*^ coding sequence is sufficient to instruct a feminization of the eye morphology. However, the observed feminization was partial, suggesting that at least another gene is also involved in sexually dimorphic eye differentiation.

### ZnF motif of Glu^F^ is involved in sexual eye differentiation

**Fig. 6:**
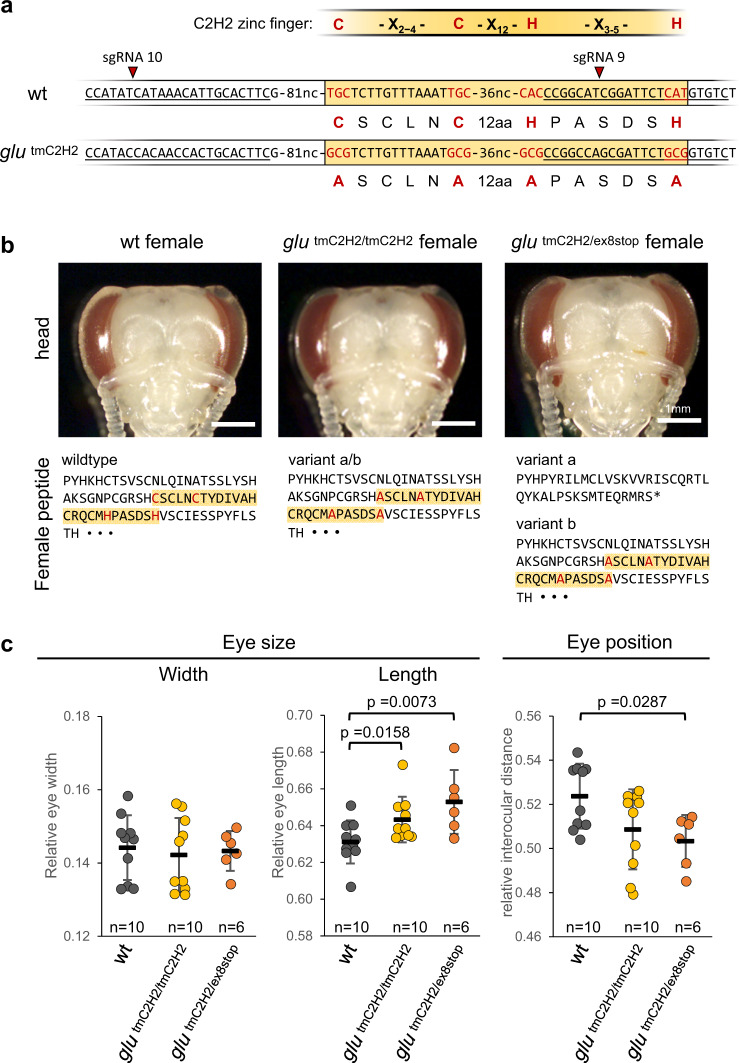
The female-specific sequence encoding the CCHH ZnF motif is required for sexually dimorphic eye differentiation. **a** Scheme of the tmC2H2 mutations that were induced via CRISPR/Cas9-mediated homology-directed repair. The CCHH ZnF core motif C-X_2−4_-C-X_12_-H-X_3−5_-H^34–36^ is shown together with the corresponding nucleotide and amino acid sequences of the wild type (wt) and the tmC2H2 allele. Red letters indicate codons and amino acids of the core CCHH motif. Silent mutations prevent the further binding of sgRNAs to their target sites. Underlined sequence: targets of sgRNA 10 and 9. C: cysteine, H: histidine, A: alanine. **b** Eye morphology of *glu* ^*tmC2H2/tmC2H2*^ (*n* = 10) and *glu* ^*tmC2H2/ex8stop*^ (*n* = 6) mutant females at pupal stage 4. The amino acids around the ZnF coding sequences are shown. The zinc finger module (ZnF) is highlighted in yellow, while the induced amino acid changes of the core motif are shown in red. **c** Relative sizes and positions of the eyes in the mutants (one tailed Mann–Whitney U test). Means and standard deviations are shown.

The key regulators shaping developmental features are often transcription factors (TFs) regulating multiple downstream genes and terminal cellular features. TFs are characterized by DNA binding domains such as CCHH zinc finger domains^28,32,33^. To examine whether the female-specific CCHH ZnF motif of Glu is such a key element of a possible TF, we altered essential positions of this motif^34–36^ and studied its role in sexually dimorphic eye development. It has previously been difficult to ascertain such motif functions in non-genetic models. Here, we employed CRISPR/Cas9-mediated homology-directed repair to replace the nucleotides encoding cysteine and histidine of the core ZnF motif with those encoding alanine (Fig. 6a) with the aim of disrupting a possible ZnF structure in females^37–39^. The homozygous double mutants (*glu* ^*tmC2H2/tmC2H2*^) showed a significantly greater relative eye length (*p* < 0.05), somewhat shorter interocular distance (*p* = 0.06) and the same eye width as the controls (Fig. 6b, c), suggesting a function of this motif in sexually dimorphic development. Moreover, the heterozygous *glu* ^*tmC2H2/ex8stop*^ females (with one allele a loss of function allele) showed a greater partial loss of female characteristics, representing an intermediate phenotype between the homozygous *glu* ^*tmC2H2/tmC2H2*^ and *glu* ^*∆ex2-8/∆ex2-8*^ mutants (Fig. 6b, c). These results demonstrate a role of the CCHH motif in sexually dimorphic eye development, supporting the notion that the *glu* gene encodes a female-specific transcription factor. However, eye width was not affected by the CCHH mutation. Since developmental regulators of the ZnF type usually have more than one ZnF domain^28,36^, we next asked whether female Glu proteins contain other ZnF motifs. We found three other possible ZnF motifs of the non-canonical type located around the CCHH ZnF motif (Supplementary Fig. 5), indicating the possibility of additional DNA binding domains.

### Sex-specific splicing and ZnF motif originated stepwise

**Fig. 7:**
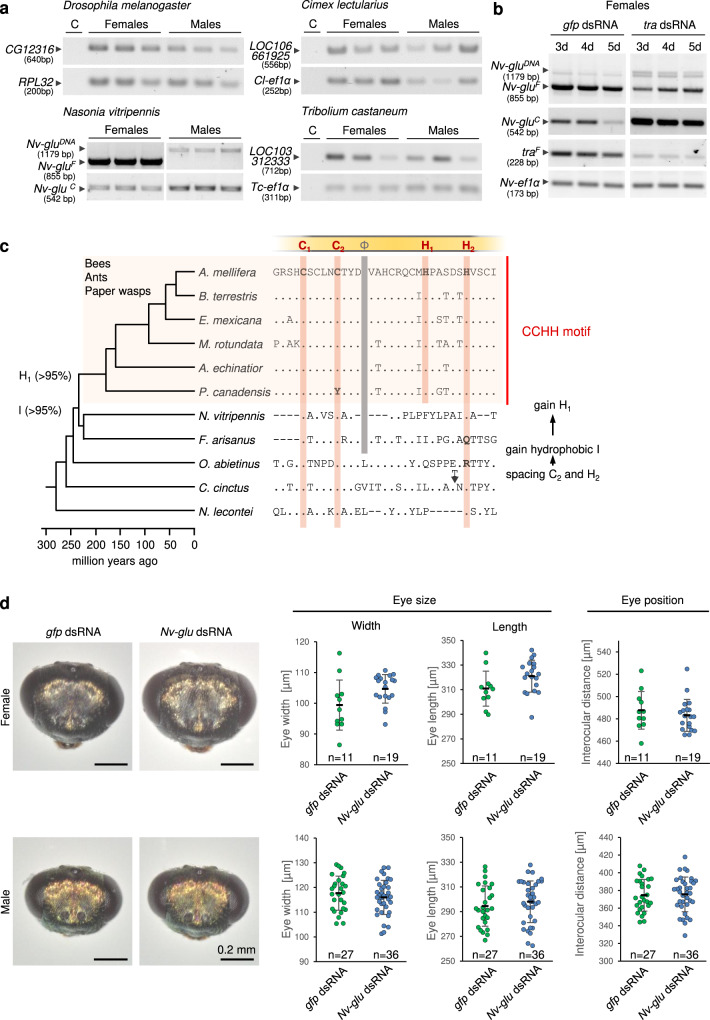
Sex-specific control, ZnF motif and sexually dimorphic function of the *glu* gene evolutionary originated within the hymenopteran insect lineage. **a** Sex-specific splice control evolved in the hymenopteran lineage. Splicing of *glu* orthologs in *D. melanogaster* (CG12316)*, C. lectularius* (*LOC106661925*), *T. castaneum* (*LOC103312333*) and *N. vitripennis* (*Nv-glu*). The sequences homologous to exon 7/8 of the honeybee were studied. Semiquantitative RT-PCR results were adjusted across three biological replicates using *ef1α* (*elongation factor 1α*) transcripts of *C. lectularius* (*Cl-ef1α*) and *T. castaneum* (*Tc-ef1α*) and the *ribosomal protein L38* of *D. melanogaster* (*RPL38*). *Nv-glu*^*F*^ of *N. vitripennis* is female-specifically spliced and encodes a female-specific peptide, while *Nv-glu*^*C*^ is common to both sexes. *Nv-glu*^*DNA*^ is the amplified genomic sequence. **b** The sex-specific splicing of *Nv-glu* in *N. vitripennis* is controlled by the *tra* gene. The knockdown of the *tra* gene was mediated by systemic RNAi. dsRNA: double-stranded RNA. *Gfp*: control dsRNA. The RT-PCR results were semiquantitatively adjusted across three pools collected after 3, 4 and 5 days (d) after treatment using *Nv-ef1α* transcripts. C: negative control for PCR. **c** The female-specific CCHH ZnF motif evolutionarily originated in hymenopteran insects. Amino acid sequence alignment of *glu* homologs and phylogenetic relationships of the corresponding species. Representative examples from major evolutionary lineages and 49 hymenopteran sequences are shown in Supplementary Fig. 7. The phylogenetic relationships and evolutionary divergence times follow Peters et al.^70^. Red small boxes: amino acids of the core motif; gray boxes: hydrophobic core residue; % values next to the nodes: inferred likelihood of changes according to the parsimony method. **d** The *glu* homolog of *N. vitripennis* is not involved in sex-specific eye differentiation. Sex-specific eye differentiation of *N. vitripennis* males and females in response to *Nv-glu* gene knockdown via systemic RNAi. Absolute rather than relative eye parameters are presented since the larval injections and dsRNA treatment alone affected the general size of the adult head (Supplementary Fig. 9). For comparison the absolute and significant values of honeybees are provided (Supplementary Fig. 10). Means and standard deviations are shown.

Since the function of *glu* as a sex-specific developmental regulator of sexually dimorphic differentiation has not been described in other species, we examined whether this function newly evolved. To gain insight into the evolutionary history of this gene, we first examined the sex-specific expression of *glu* homologs in species derived from major insect lineages (Supplementary Fig. 6). In the dipteran insect *D. melanogaster*, the beetle *Tribolium castaneum*, and the hemipteran insect *Cimex lectularius* (bed bug), *glu* homologs were not sex-specifically spliced or transcribed (Fig. 7a). However, in the hymenopteran jewel wasp *Nasonia vitripennis*, the transcripts of the *glu* homolog (*Nv-glu*) were sex-specifically spliced with a female-specific transcript and a transcript common to females and males (Fig. 7a and Supplementary Fig. 6). We next examined whether this sex-specific splicing in the jewel wasp was controlled by the *tra* gene (which is the ortholog of the honeybee *fem* gene). The knockdown of the *tra* gene in *N. vitripennis* females using systemic RNAi resulted in a reduction in the female-specific *Nv-glu* transcript and a large increase in the common transcript relative to *gfp* dsRNA-treated controls (Fig. 7b). This result suggests that the *tra* gene controls (either directly or indirectly) the female-specific splicing of the *glu* ortholog in *N. vitripennis*. We conclude from the maximum parsimony inference of these results that the sex-specific splicing of *glu* by *fem*/*tra* has evolutionary emerged in the hymenopteran insect lineage.

Our mutational studies revealed that the female-specific CCHH ZnF motif is a key element of the sexually dimorphic differentiation of the compound eye in honeybees. To determine whether the CCHH ZnF motif de novo originated by amino acid replacements (it is, for example, absent in *D. melanogaster*), we inferred the likelihood of different ancestral states of the amino acids using the maximum parsimony method from a set of *glu* homolog sequences from 49 hymenopteran species. The identified ancestral states suggest that the CCHH ZnF motif originated within the Aculeata lineage after splitting from the parasitoid wasp lineage, which includes the jewel wasp *N. vitripennis* (Fig. 7c and Supplementary Fig. 7). We found that the CCHH ZnF motif evolved via a series of changes that could be reconstructed from the ancestral states and the phylogeny of the 49 sequences (Fig. 7c and Supplementary Fig. 7). The sequence of the evolutionary changes was as follows: (i) gain of the required spacing between the 2^nd^ cysteine and 2^nd^ histidine via insertions/deletions; (ii) gain of the hydrophobic amino acid isoleucine (I), a core residue of the canonical motif, by point mutation^28,36^; and (iii) gain of the amino acid histidine (H), which completed the CCHH ZnF motif. These results suggest that the CCHH ZnF motif evolved de novo via a series of mutations in the coding sequence.

The *glu* homolog in *D. melanogaster* (CG12316) is not sex-specifically spliced and has no annotated phenotype^40–42^, raising the question of whether the function of *glu* as a sex-specific developmental regulator newly evolved. To obtain evidence regarding the evolutionary origin of this function, we examined the role of *Nv-glu* in *N. vitripennis* using systemic RNAi (Supplementary Fig. 8). This study in *N. vitripennis* was informative in that respect, since *Nv*-*glu* may represent a possible intermediate evolutionary state of the past, in which sex-specific splicing was gained while a CCHH ZnF motif was still absent. Additionally, the interocular distance of males and females was previously shown to be sexually dimorph in *N.vitripennis*^18^. The width, length and intraocular distances of the compound eyes did not differ between *Nv-glu* knockdown and control individuals, in both females and males (Fig. 7d). These results from systemic RNAi experiments suggest that the *Nv-glu* gene does not control sexually dimorphic eye differentiation in the jewel wasp. Collectively, these comparative results led us to the conclusion that the role of *glu* as a regulator of sexually dimorphic eye development recently evolved within hymenopteran insects and this in the lineage leading to honeybees.

## Discussion

### The glu gene operates as a genetic switch

A central interest of developmental biologists is to understand how sex determination signals are integrated with the general developmental program. This is a particularly intriguing issue, as the differences between the sexes can be manifold, and the sexual dimorphisms are astonishingly diverse among organisms suggesting rapid divergence. We have now characterized with the *glu* gene another regulator of sexual development and elucidated its molecular mechanism of control, providing further understanding of how sexually dimorphic structures are formed.

**Fig. 8:**
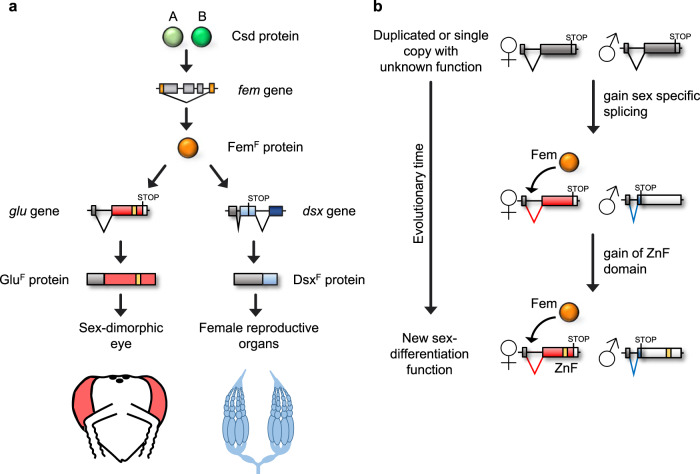
Developmental and evolutionary mechanisms underlying sexually dimorphic differentiation. **a** The region-specific roles of the *glu* and *dsx* genes in sexually dimorphic differentiation of female honeybees. **b** The sequence of the evolutionary steps giving rise to a genetic switch for sexually dimorphic differentiation. Boxes denote the exons. Female-specific parts of the ORF are shown in red, male-specific parts are displayed in blue, and common parts are shown in dark gray. The zinc finger motif (ZnF) is indicated in yellow.

We showed that sexually dimorphic eye differentiation in honeybees is partially regulated by the *glu* gene, a sex-specific developmental regulator that has not previously been reported. The *glu* gene acts as a sex-specific genetic switch (Fig. 8a). Sex-specific activity is provided by the female- and male-specific transcript splicing, which is controlled by the *fem* gene an ortholog of the *tra* gene^25,26^. The Fem proteins, which are limited to females, direct the use of an alternative splice acceptor site in exon 8. These female-specific transcripts encode Glu^F^ proteins (1466 to 1572 amino acids) with ZnF domains. In the absence of Fem proteins in males, another splice acceptor site in exon 8 is employed, producing an early stop codon in *glu* transcripts. The predicted male protein with no ZnF domain is 226-332 amino acids long. However, the lack of *glu* transcripts in male pupae and adults suggests that the male spliced transcript has no function. Possibly, this lack of transcript is due to the early stop codon, which may induce nonsense-mediated decay of the transcript^31^. According to our functional studies, the most obvious regulation of this genetic switch is that the expression of the female-specific ZnF domain-containing protein (Glu^F^) provides activity exclusively in females. The male transcripts produce either a non-functional protein or no protein.

The Glu^F^ protein is likely a transcription factor of the ZnF domain type, which regulates a feminization of eye morphology (Fig. 8a). Male-specific differentiation results from the regulatory default state of this genetic switch. Our studies showed an intersexual phenotype with different degrees, suggesting that *glu*^*F*^ is only partly responsible for regulating the overall sexual eye structure and is mainly responsible for the sexually dimorphic lens facet size. This suggests that other sex-specific regulated genes must be involved in shaping sexually dimorphic eye differentiation. This could be other sexually regulated developmental genes and/or genes with general function such as cellular proliferation, which can affect eye size and shape. Sex-specific proliferation of stem cells has been demonstrated in *Drosophila*, which is responsible for a larger size of the female midgut organ^43^.

### The glu gene selectively regulates sexual eye morphology in a region-specific manner

Previous studies have shown that DM domain genes are central, conserved regulators of sex-specific development. DM domain gene homologs in different animal phyla instruct external sexual morphology, such as the tail morphology of *C. elegans*^6,7^, the male-specific antenna and copulatory thoracic hook of the crustacean *D. magna*^8^, the sex combs of *D. melanogaster*^9,10^ and the exaggerated horn structures of some beetles^11–13^. The DM domain genes thereby act as a sex-specific genetic switch that produces distinct activities in males versus females. In insects, sex-specific activities are established via splicing. Splicing mediated by *fem*/*tra* homologous genes leads to the expression sex-specific Dsx proteins with a shared DM domain but different C-termini in many insects^3,44,45^.

Two central questions have been (i) how DM domain genes can instruct sex-specific structures in different body parts and (ii) how widely conserved DM genes can instruct the formation of newly evolved sexually dimorphic structures that were previously absent in an animal lineage. Two mechanisms have been suggested, which resulted mainly from work focusing on the *dsx* gene in insects and the genus *Drosophila*^2,10,12,46–50^. A mechanism that explains the sexually dimorphic structures in different body parts suggests that local expression of the *dsx* gene provides the region-specific sexual instruction. For example, the male-specific sex combs are confined to the foreleg of *Drosophila* and are induced by local *dsx* expression in that tissue^9,10^. A mechanism explaining the evolutionary origin of a new sexual dimorphism suggests that evolutionary gains and modifications of the *cis*-regulatory elements of *dsx* target genes establish altered gene regulations for new characteristics^46,51^. For example, the extension of abdominal body pigmentation in *D.melanogaster* males can evolve from evolutionary gains and modifications of these *cis*-regulatory elements^46,51^.

The results of our study on *glu* now suggest other mechanisms underlying local formation and evolutionary origin of a sex-specific dimorphism. We showed that *glu* in honeybees is a newly evolved sex-specific developmental regulator that is expressed in a tissue-specific manner and selectively regulates eye feminization (Fig. 8). In honeybees, *dsx* regulates sexual reproductive organ development^21^ and not the sexual differentiation of the head or eye (Supplementary Fig. 1). These results suggest a mechanism, in which sexual dimorphism in distinct body parts is regulated by different sex-specific developmental regulators (*glu* and *dsx*). They operate in parallel, but in different regions of the body via tissue-specific expression (Fig. 8a). Furthermore, the evolution of *glu’s* function also provided insight into evolutionary mechanisms underlying a possible origin of a sexually dimorphic structure. We showed that the evolutionary origin of sex-specific expression (via gain of sex-specific splicing) plus the gain of molecular function (via the origin of a ZnF motif) led to a new sex-specific developmental regulator for sexual dimorphism.

### Sex-specific expression evolved before the function of glu in sexual development

The origin of sexually dimorphic traits remains a central issue in evolutionary biology. The question remains, how such new sex-specific regulation for dimorphic structures might originate. Comparative functional and sequence studies of the *glu* gene suggest that this function originated in a sequence and two steps (Fig. 8b). In the first step, *glu* was recruited to the sex determination pathway, which established the sex-specific genetic switch and expression. We demonstrated this gain of sex-specific control via the origination of the *fem*-dependent splice control over *glu* transcripts, which possibly occurred after the splitting of the hymenopteran lineage from other major insect lineages. In the second and following step, the *glu* gene gained its sex-specific eye differentiation function within the hymenopteran insects and this in the lineage leading to the honeybee (Fig. 8b). It was previously shown that *dsx* is responsible for male head patterning and eye size in the genus *Nasonia*^18^, while we showed that *glu* has no influence on eye size in *N.vitripennis*. Furthermore, we demonstrated by examining the evolution and the function of the ZnF motif that the role of this domain for sex-specific eye differentiation newly evolved after the gain of sex-specific expression.

The sequence of these changes provides insight into how novel sexually dimorphic structures can originate during evolution. We observed a sequence of evolutionary changes—gain of sex-specific expression followed by an origin of sex-specific developmental function—, which has long been predicted by theory, but has to our knowledge not been demonstrated^52–54^. We found that the evolutionary gain of *glu* sex-specific expression was the initial step that limited some mutations to the coding sequence of the female protein. We further demonstrated that these mutations in the female coding sequence were involved in the formation a new ZnF motif that has female-limited developmental function. Thus, our findings demonstrate that the gain of sex-specific expression is a molecular path through which new developmental regulators for sexual dimorphism can evolutionarily originate.

## Methods

### Animal sources

The honeybees used in this study were derived from feral *Apis mellifera carnica* colonies. Female embryos (which are diploid) were collected from eggs laid by naturally mated queens. Haploid male eggs were collected from non-mated queens treated with CO_2_, which induces the laying of unfertilized eggs. Embryos were collected using a Jenter egg collection box (Jenter Queen Rearing Kit, Karl Jenter GmbH, Frickenhausen, Germany) and were either injected or left in the incubator at 34 °C until the targeted stage^55^. Wild type pupae and adults were collected from bee colonies. Wild type *Cimex lectularius* adult males and females were purchased from Insect Services GmbH (Berlin, Germany). *Drosophila melanogaster* adults from the isogenic strain ^w1118^ were a gift from Hermann Aberle (Heinrich-Heine University Düsseldorf, Germany). Adult *Tribolium castaneum* males and females were a gift from Gregor Bucher (University of Göttingen, Germany). The laboratory AsymCx strain of *Nasonia vitripennis* was cured of *Wolbachia* infection and continuously reared on *Calliphora* sp. hosts at 25 °C. Male and female wasps were separated based on the sex-specific forewing size before eclosion. Male-only offspring were generated by offering hosts to virgin females. To produce female offspring, a virgin female was paired with a single male and allowed to mate for one day. Two hosts per day were provided to individual females to initiate oviposition.

### DNA and RNA preparation and RT-PCR

Genomic DNA was isolated from honeybee L1 larva, queen leg or pupal hindleg tissue with the innuPREP DNA Mini kit (Analytik Jena, Jena, Germany). RNA from dissected honeybee tissue and *D. melanogaster, T. castaneum* or *C. lectularius* adults (three pooled individuals of each sex) was isolated using the TRIzol method (Thermo Fisher Scientific, Braunschweig, Germany). RNA from larval stage 1, embryos or from pools of embryos was isolated using the innuPREP DNA/RNA Mini kit (Analytik Jena, Jena, Germany). cDNA was synthesized using the RevertAid First Strand cDNA Synthesis Kit and oligo dT or random hexamer primers (Thermo Fisher Scientific, Braunschweig, Germany). Subsequently, the second strand was synthesized by using DNA Polymerase I^21^. The purification of cDNA was performed using the EZNA Cycle Pure kit (Omega Bio-Tek. Inc., Norcross, USA). RNA from *N. vitripennis* was extracted using ZR Tissue & Insect RNA MicroPrep™ (Zymo Research, Freiburg, Germany) with on-column DNase I treatment (Thermo Fisher Scientific, Braunschweig, Germany). In this case, cDNA was synthesized using the SensiFAST™ cDNA Synthesis Kit (Bioline, London, England).

Phusion™ High-Fidelity DNA Polymerase (Thermo Fisher Scientific, Braunschweig, Germany) was used for RT-PCR if the amplicons were to be subsequently sequenced. Otherwise, GoTaq® G2 Flexi DNA Polymerase (Promega, Walldorf, Germany) was used. PCR was performed with a standard temperature profile^56^. For the semiquantitative RT-PCR, the amount of template and the number of cycles were adjusted according to reference gene *elongation factor 1-alpha* (*Nv-ef1α, N. vitripennis; Cl-ef1α, C. lectularius; ef1α, A. mellifera*) and *ribosomal protein L32* (*RPL32; D. melanogaster*) to exclude saturation and allow adjustment across samples. The employed oligonucleotides (Eurofins Genomics, Ebersberg, Germany) with their sequences are listed in the Supplementary Data 1. Uncropped and unprocessed scans of agarose gels are provided in the source data file.

### Sequence analyses

The exon structure of *glu* (*LOC552468*) in *A. mellifera* was determined using RNAseq data^27^ and the amplicon sequences obtained via RT-PCR using RNA derived from 25 to 40 h old male and female honeybee embryos and brain tissues of female pupae. Domain searches were conducted with PROSITE (https://prosite.expasy.org/)^57^, InterPro (https://www.ebi.ac.uk/interpro/)^58^, and the Pfam database (https://pfam.xfam.org)^59^. Homologous proteins were identified by BLASTP searches of the NCBI database with significant similarity (https://blast.ncbi.nlm.nih.gov/Blast.cgi). The identified genes were *LOC100678462* (*Nv-glu*; *N. vitripennis*)*, LOC106661925* (*C. lectularius*), *LOC103312333* (*T. castaneum*) and *CG12316* (*D. melanogaster*). Homologous positions and possible boundaries of coding exons were deduced by employing the protein sequences. Ancestral states were inferred using the maximum parsimony method^60^. Evolutionary sequence analyses were conducted using MEGA6^61^. The sequences presented in Fig. 7c are as follows: *A. mellifera* XP_026299695.1; *Bombus terrestris* XP_012165493.2; *Eufriesea mexicana* OAD55885.1; *Megachile rotunda* XP_012146010.1; *Acromyrmex echinatior* XP_011060010.1; *Polistes canadensis* XP_014601325.1; *N. vitripennis* XP_003425013.1; *Fopius arisanus* XP_011314024.1; *Orussus abientinus* XP_012279333.1; *Cephus cinctus* XP_015598325.1; *Neodiprion lecontei* XP_015517082.1.

### CRISPR/Cas9-mediated mutagenesis in the honeybee

Target sites for sgRNAs were identified with Benchling software (https://benchling.com/). The target sites were 20 nt long, started with a 5’ guanidine and showed at least three mismatches to alternative targets in the genome, the possible off targets. PCR generated DNA fragments containing a T7 RNA polymerase transcription start site, the target sequence and the Cas9 protein binding sequence. sgRNAs (sequences listed in Supplementary Data 1) were synthesized using the DNA fragment as a template and a RiboMax Kit (Promega, Walldorf, Germany). The sgRNAs were purified using a MEGAclear Kit (Thermo Fisher Scientific, Braunschweig, Germany) and mixed at a molar ratio of 2:1 with 500 ng/µl Cas9 protein (New England Biolabs, Frankfurt am Main, Germany). For targeted mutation, 15–20 pg dsDNA per 400 pl injection mixture was added. dsDNAs were produced according to the provided sequence (Eurofins Genomics, Ebersberg, Germany) with 250 bp flanking homologous sequences. The sequences were amplified by PCRs and purified using the EZNA Cycle Pure kit (Omega Bio-Tek Inc., Norcross, GA) prior to injection. Honeybee embryos (0–1.5 h old) were injected with 400 pl sgRNA/Cas9 mixture and were kept in the incubator until the larvae hatched^55,62^. The rearing of larvae was performed on 170 mg of a worker diet (w/v in sterile water: 50% royal jelly, 15% glucose, 15% lactose, 1% yeast extract)^63^ at 34 °C under 94% humidity until larval stage 5. The larvae were transferred to Petri dishes equipped with filter paper and maintained until pupal stage 4 at 34 °C under 75% humidity^64^. For the analysis of *glu* ^*ex7-8 F*^ males, injected female larvae were raised to queens, and male egg laying was induced by the CO_2_ treatment of virgin queens^55^. Male offspring were collected after eclosing and kept in small hives in an incubator with young worker bees. Phenotyping was performed on adult males 1–16 days after eclosing. The injected individuals were screened for the desired mutations. The target site of the mutations was amplified (DNA for *glu* and cDNA for the *fem* gene; Supplementary Data 1)^21^. In the first step, individuals were prescreened using length differences in the amplicons. Individuals with *glu* ^*∆ex2-8/∆ex2-8*^ deletions and *glu* ^*ex7-8 F*^ mutations were identified by resolving amplicons by gel electrophoresis. For other mutations, fragment length analyses were performed by capillary gel electrophoresis using hexachlorofluorescein-labeled primers for PCR^21^. Mutations in *glu* ^*ex7-8 F*^ were further verified by nucleotide sequencing. We identified *glu* ^*tmC2H2*^ mutations by the restriction digestion of amplicons with the enzyme *PdiI* (Thermo Fisher Scientific, Braunschweig, Germany) and gel electrophoreses. The detected length variants were further validated based on the nucleotide sequences obtained via the DNAseq of amplicons. Index PCR were run using the Nextera XT Index Kit (Illumina, San Diego, USA), and amplicons were purified with Agencourt AMPure XP beads (Beckman Coulter, Brea, USA). Library preparation and sequencing (2 × 250 bp reads) using the MiSeq Reagent Kit v2 (500 cycles; Illumina, San Diego, USA) were performed by the Center for Biological and Medical Research (BMFZ, Heinrich-Heine University, Germany) following Illumina protocols. A minimum of 78,000 reads per sample were generated on an Illumina MiSeq system (Illumina, San Siego, USA). Raw sequences were processed and analyzed using the Galaxy online toolset (http://usegalaxy.com)^65^. Low-abundance sequences that were unrelated (<5% of reads) were excluded^21^.

### Knockdown of *tra*^*F*^ and *Nv-glu* in the jewel wasp

A MEGAscript RNAi Kit (Thermo Fisher Scientific, Braunschweig, Germany) was used to produce dsRNAs (*tra*^*F*^*, Nv-glu, gfp*: green fluorescent protein-derived sequence). *Nv-glu* dsRNA targeted the common part of the transcript, which is present in both sexes. The *gfp* sequence was amplified from the vector pOPINEneo-3C-GFP, which was a gift from Ray Owens (Addgene plasmid # 53534; http://n2t.net/addgene:53534; RRID: Addgene_53534). dsRNA *Nv-tra*^*F*^ was injected into 4th-instar female larvae, while dsRNA *Nv-glu* was injected into 2nd-instar male and female larvae, both at a concentration of 4000 ng/μl. Food dye was added to the dsRNA solution (1:9 v/v) to guide injection. Injection into *N. vitripennis* larvae^66^ was carried out using the FemtoJet® 4i injector (Eppendorf, Hamburg, Germany). Injected 2nd-instar larvae were transferred to their foster hosts (6-8 larvae per host), which were placed on 1X PBS plates^66^. dsRNA *Nv-tra*^*F*^-treated samples were collected 3, 4, and 5 days after injection and pooled (*n* = 4 to 5). dsRNA *Nv-glu* samples were collected at the adult stage for phenotyping.

qRT-PCR was performed with 3 to 7 replicates on an CFX96^TM^ Real-Time System (Bio–Rad, Hercules, USA) following the procedure of the SensiFAST^TM^ SYBR® No-ROX Kit (Bioline, London, England). *Nv-glu* qPCR primers were designed outside the dsRNA target region. Values were analyzed using CFX Manager 3.1 software (Bio–Rad, Hercules, USA).

Relative expression levels were calculated using LinRegPCR software (LinRegPCR, 2017.1.0.0, HFRC, Amsterdam, Netherlands)^67^. *Nv-glu* knockdown was performed in two separate experiments (Supplementary Fig. 8).

### Phenotyping

Heads were dissected from honeybee pupae and photographed using an S8 APO binocular microscope (Leica, Wetzlar, Germany) with a UI-1240LE-C-HQ camera (IDS, Obersulm, Germany) and uEye Cockpit (part of IDS suite v4.92, IDS, Obersulm, Germany) software. Images for dorsal lens facet diameter measurements were taken with a Canon Eos 6d Mark II camera and Canon MP-E65 mm 1:2,8 1–5× Marco objective (Tokyo, Japan). Images of *N. vitripennis* adult heads were obtained using a Dino-Lite Edge 5MP Digital Microscope and DinoCapture 2.0 software (Dino-Lite, Almere, The Netherlands). Head parameters (head width, head length, eye width, eye length and interocular distance) were measured as schematically presented (Supplementary Fig. 11). In *glu* ^*ex7-8 F*^ males and controls, lens facet diameter was measured in the frontal-dorsal region of the eye, where facets in males should be largest^24^. The mean of 15 randomly measured lens facet diameters per individual was used for analysis. Length measures were performed using ImageJ (National Institute of Mental Health, USA).

### Statistics and reproducibility

Data statistical analyses were performed using SigmaPlot 14.0 (Systat, San Jose, United States of America). The phenotype data comparison was analyzed via two-tailed (or one-tailed in Fig. 6c) Mann–Whitney U tests. For expression level comparisons in *N. vitripennis* Student’s t-tests were applied. The plots of the data show the means and standard deviations (Figs. 5c, f, 6c, 7d and Supplementary Figs. 1, 9, 10) or standard errors (Supplementary Fig. 8). No data were excluded except for knockdown phenotype data of *N*. *vitripennis*, in which the injection procedure alone produced extreme outliers and variations for the size phenotypes of the head (Supplementary Fig. 9). We used 1.5 times the standard deviation as criterion to remove such outliers^68^ from head width or length data from both the control and treated group following the procedure (Fig. 7d and Supplementary Fig. 9). Sample-size calculations were not performed. Instead, sample size was chosen based on similar previously published studies of honeybee development. The phenotype of each single insect derived from independent mutational or knock down events. These data points represent independent biological replicates. The investigators were blinded; they had no knowledge whether the insect belongs to the treatment/mutation or control group during phenotyping.

### Reporting summary

Further information on research design is available in the Nature Portfolio Reporting Summary linked to this article.

## Supplementary information

Supplementary Information

Description of Additional Supplementary Files

Supplementary Data 1

Reporting Summary

## Data Availability

The authors affirm that all data necessary for confirming the conclusions of the article are presented within the article, in the figures, supplementary information and data or in the source data file. Previously published RNA-seq data analyzed here is available in NIH GEO under accession number GSE159387. Sex specific cds sequences of the glu gene were deposited in the data base NCBI under the accession codes OQ116780 and OQ116781 (https://www.ncbi.nlm.nih.gov/gene). The databases PROSITE (https://prosite.expasy.org/)^57^, InterPro (https://www.ebi.ac.uk/interpro/)^58^, Pfam (https://pfam.xfam.org)^59^ and BLASTP tool of the NCBI database (https://blast.ncbi.nlm.nih.gov/Blast.cgi) used in this study are accessible online. Source data are provided with this paper.

## Source data

Source Data
