# Supplementary material for: The function and evolution of a genetic switch controlling sexually dimorphic eye differentiation in honeybees
Source: Nat Commun. 2023 Jan 28;14:463. doi: 10.1038/s41467-023-36153-4 (PMC9884244; doi:10.1038/s41467-023-36153-4)
Supplement: Supplementary file 1 — Supplementary Information [file 41467_2023_36153_MOESM1_ESM.pdf]

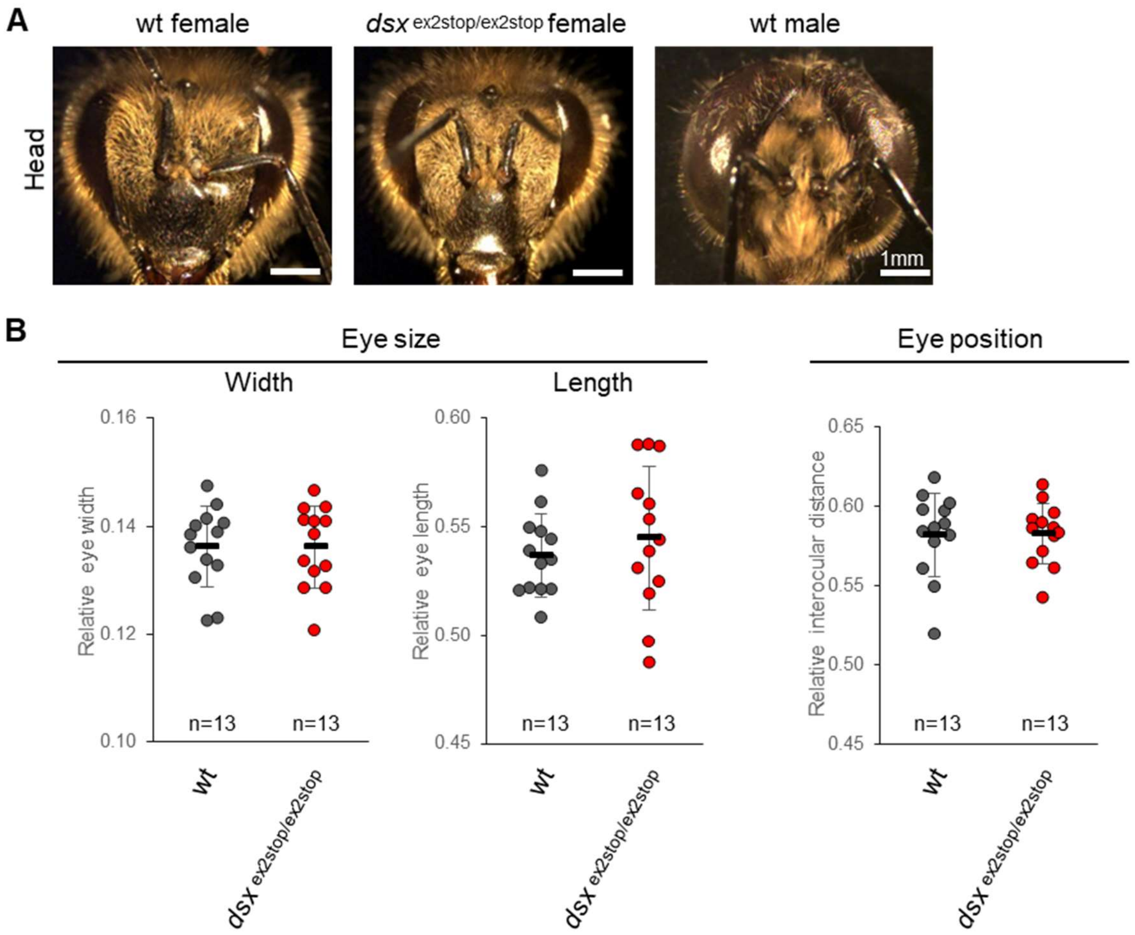

Supplementary Figure 1: Head morphology in genetic *dsx* ex2stop/ex2stop females. (A) The adult heads with the compound eyes of wild type (wt) females, *dsx* ex2stop/ex2stop females and wild type males are shown. *dsx* ex2stop/ex2stop biallelic mutations in exon 2 of the *dsx* gene were produced as described (Roth et al., 2019). (B) Relative eye width (eye width relative to head width), eye length (eye length relative to head length) and eye position (interocular distance relative to head width) of *dsx* ex2stop/ex2stop mutant and wild type females. Values do not significantly differ (two-tailed Mann-Whitney U test). Means and standard deviations are shown.

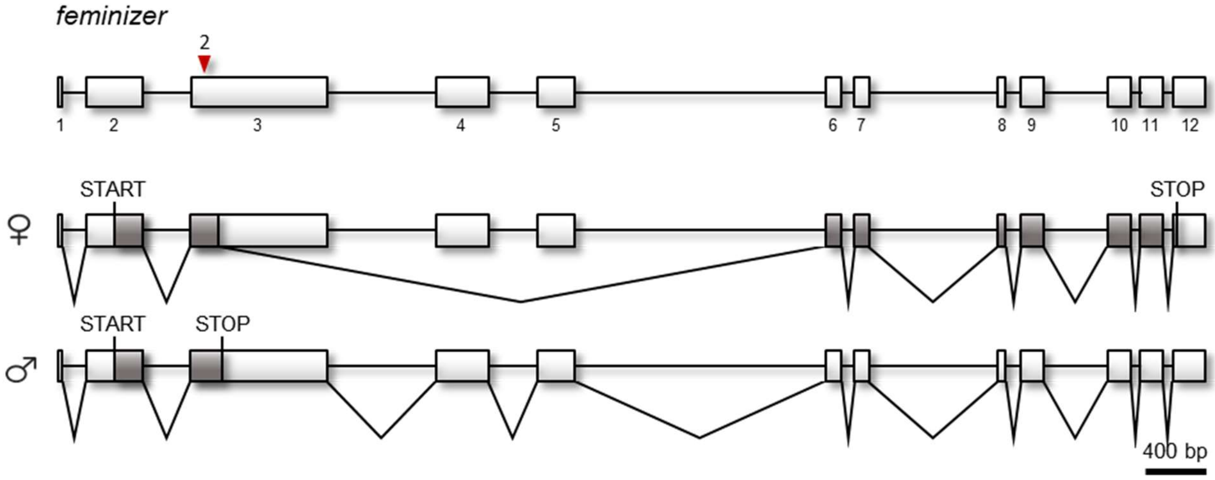

Supplementary Figure 2: Genomic organization of the *fem* gene with its female- and male-specific splice forms. Boxes indicate the exons and gray fill indicates the open reading frame (ORF). The red arrow indicates the position of the target site for sgRNA 2.

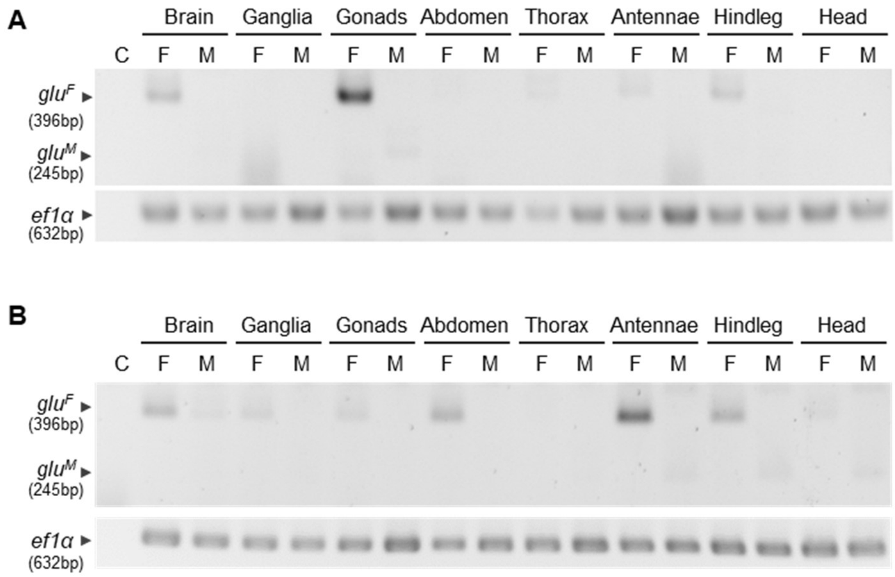

Supplementary Figure 3: The male splice variant is very low abundant or absent and not consistently detected in male pupae (A) and adults (B). Female- and male-specific fragments are amplified using a single primer combination (Supplementary data). The amplicons from one of three analyzed biological replicates are shown.

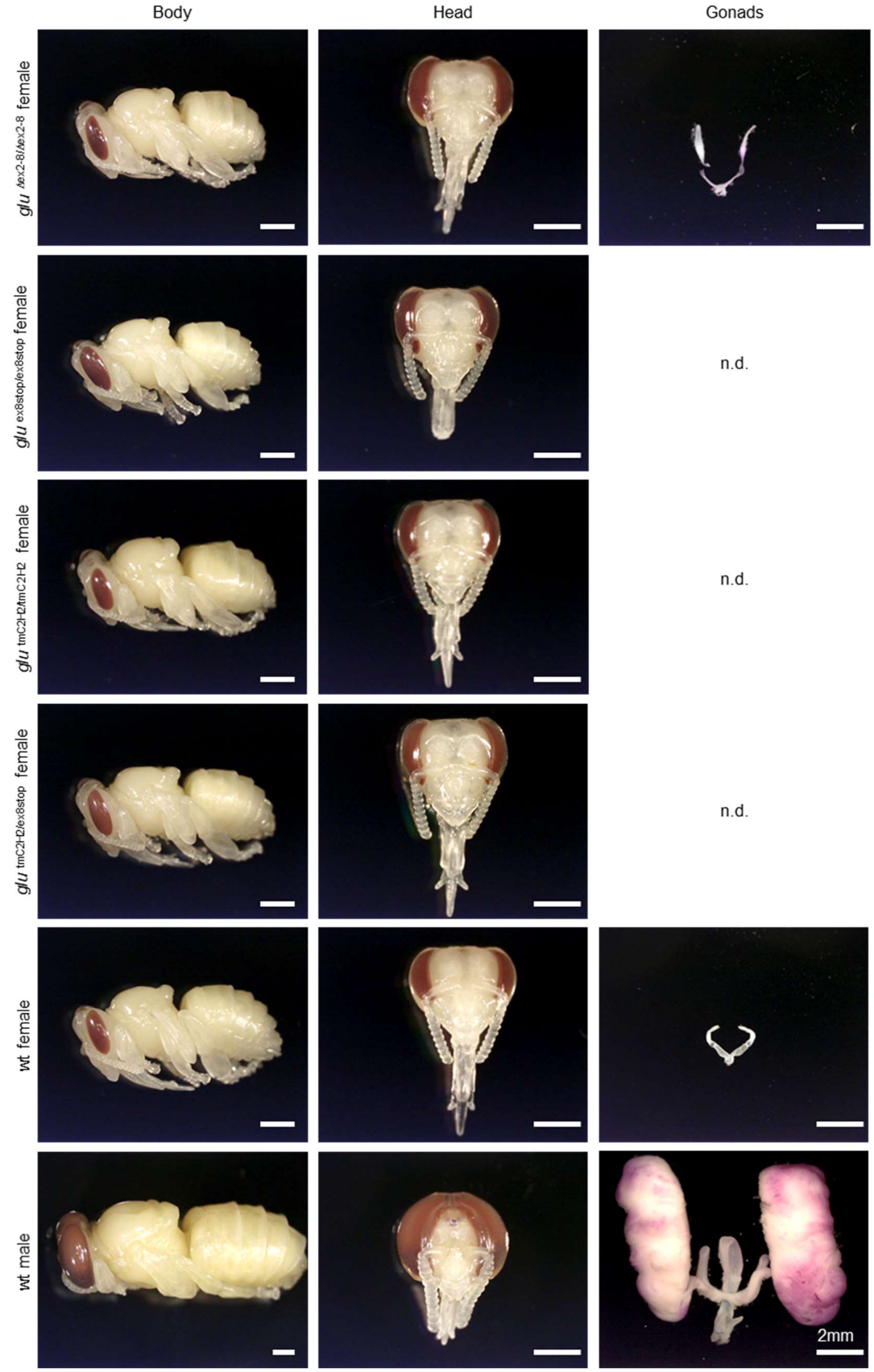

Supplementary Figure 4: The outer morphology of the genetic *glu* $^{\Delta ex2\text{-}8/\Delta ex2\text{-}8}$, *glu* $^{ex8stop/ex8stop}$, *glu* $^{tmC2H2/tmC2H2}$, *glu* $^{tmC2H2/ex8stop}$ females (full view). Female *glu* mutants showed no alterations in the macroscopic morphology of the gonads and the body compared to wild type (wt) females, except for the eyes. The eye phenotype is presented in detail in Figure 3.

...HMASNCAIPGFSGIHSHPREVDSQHPLHIPATHPTTLPAIYGNCADNTELCVPYHKHCTSVSCNLQINATSSLYS
HAKSGNPCGRSHCSCLNCTYDIVAHCRQCMHPASDSHVSCIESSPYFLSTHSSVQSPAVQEHDRAKNEVIEKLYDD
QLLCKIEKNLLQNNSLEKLEVQCDSERMFNKAAENKLPLKKRLKAHAMAYGEVQIKAKNVDNYPAMPMMSIAALEA
LDNTRKGSDQIVKSEYEVSVGKKEESHNDYHCSSNLIRRNYYKDMHVANSHQNLAKENTRKIECQFRSTNNQTDNT
ICQESCLQRTVKTSAQRKEINLSDVASLKQFDIEPMEQEGTYKKIKKTQSPLRQTRSSKRNVPKVNYSYTDVDPEW
NPSGESKRKRKKTSR*

Supplementary Figure 5: Non-canonical ZnF motifs of the female-specific peptide. Possible domains of the non-canonical zinc finger type are underlined. Red letters: amino acids relevant to zinc ion complexion, H and C. Yellow box: the position of the canonical CCHH zinc finger module.

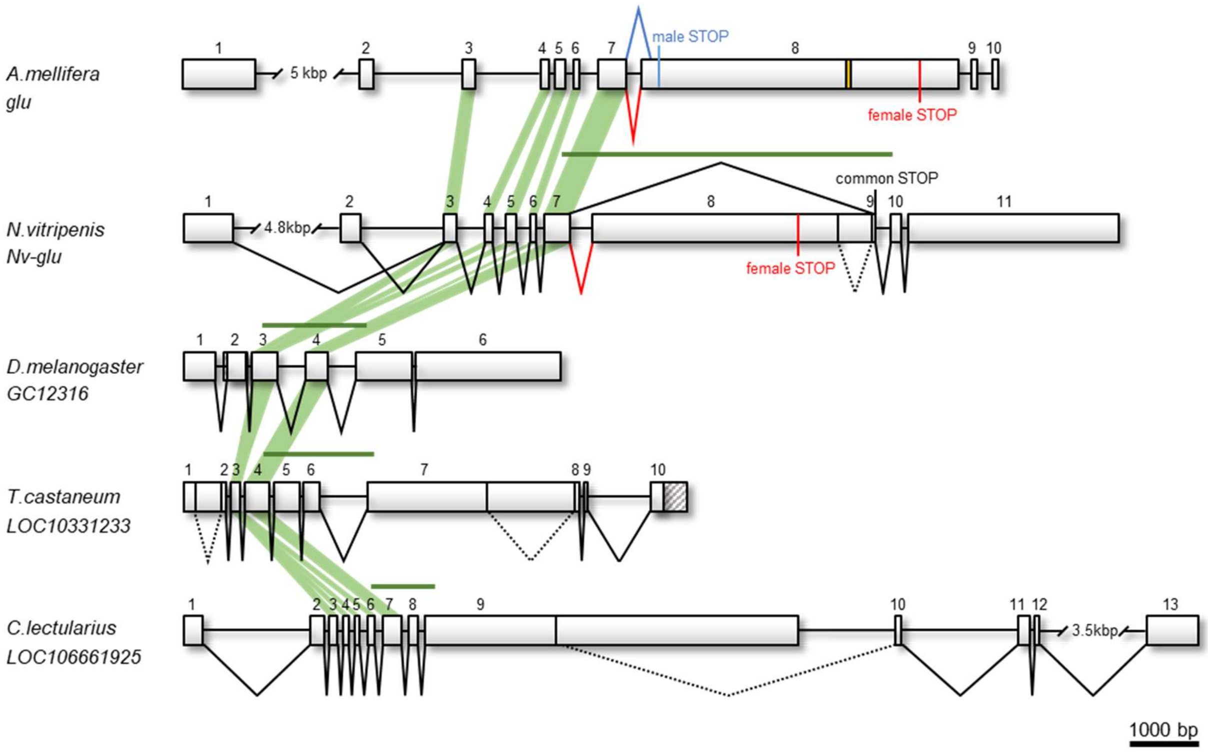

Supplementary Figure 6: Exon structure of *glu* homologs in different insect species. Exon structures of *Nv-glu, GC12316*, *LOC10331233* and *LOC106661925* are depicted according to the gene model at the NCBI database and the sequence information deposited therein. Sex-specific splice junctions are marked in red (female) and blue (male). Not sex-specific, alternative splice junctions are shown as dotted lines. The splice junctions tested for sex-specific splicing are marked with a green line above the exons. The homologous parts of the sequences are marked with interconnected green shadings.

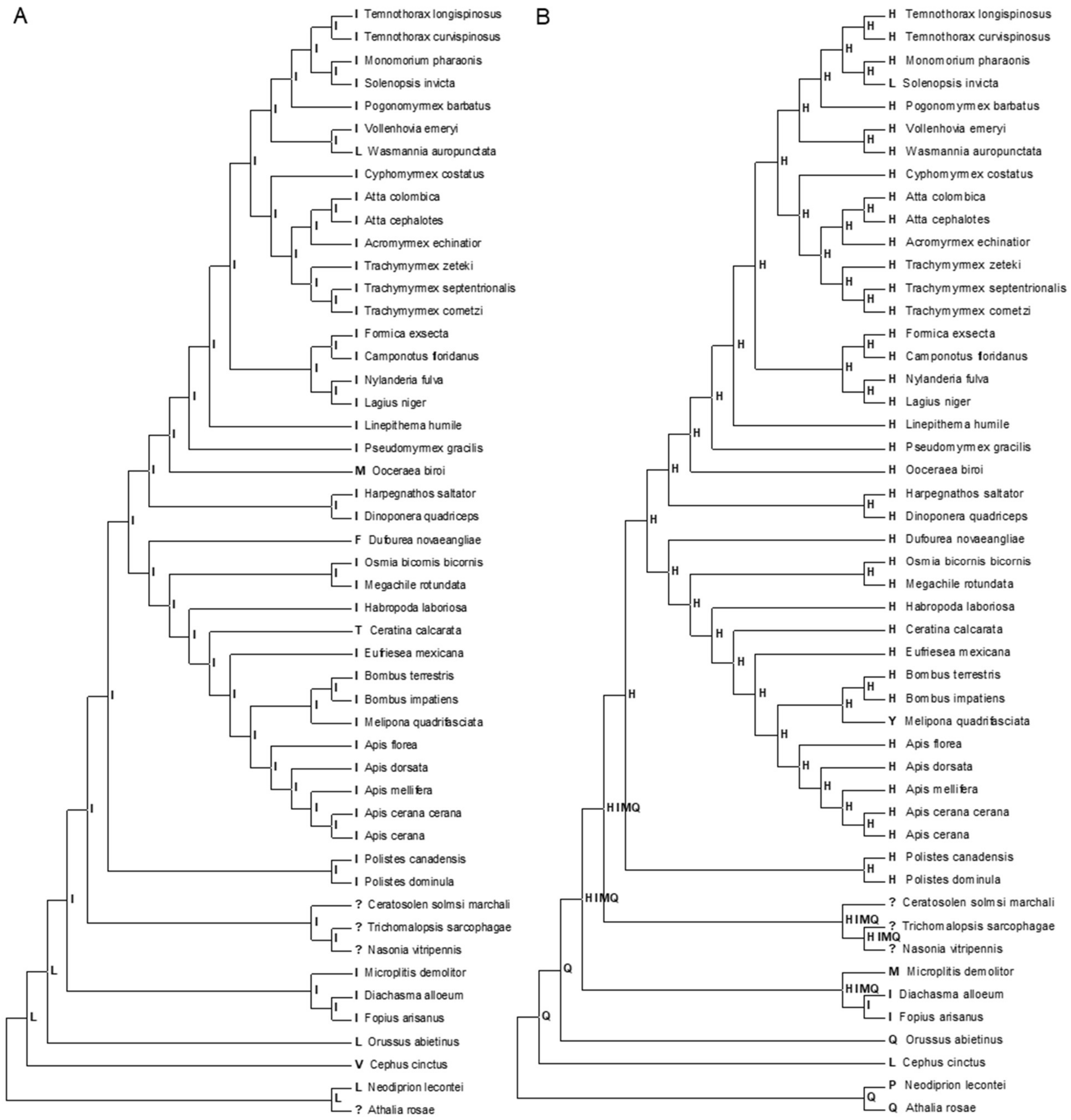

Supplementary Figure 7: Inferred evolutionary changes of amino acids at core sites of the CCHH ZnF motif. Ancestral states were inferred using the maximum parsimony method (Eck and Dayhoff, 1966). The tree from 49 sequences shows a set of possible amino acids (states) at each ancestral node based on their inferred likelihood at (A) the hydrophobic isoleucine at the core position and (B) the 2nd histidine of the canonical CCHH motif. The set of states at each node is ordered from most likely to least likely, excluding states with probabilities below 5%. Evolutionary analyses were conducted in MEGA6 (Tamura et al., 2013). There was a total of 129 positions in the final dataset. The phylogenetic relationships of the species are derived from Peters et al. (2017), Munro et al. (2011) and Moreau et al. (2006).

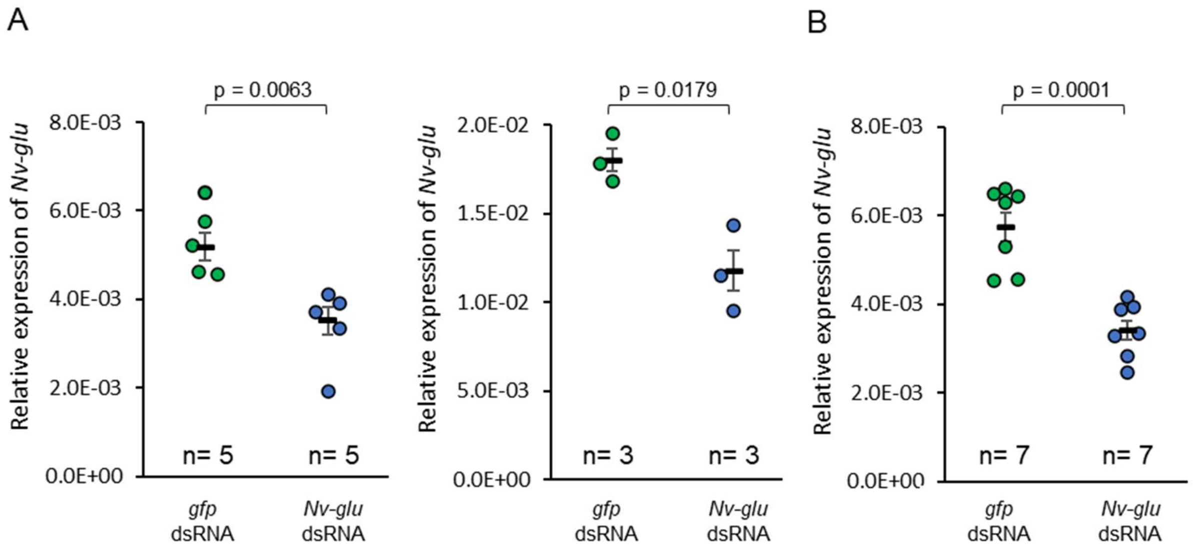

Supplementary Figure 8: Relative expression levels of *Nv-glu* in male (A) and female (B) *N. vitripennis* pupae after larval dsRNA injections. Individuals treated with *gfp* dsRNA were used as a control. Expression levels are relative to *Nv-ef1a* expression. dsRNA treatment was performed for males in two separate experiments (A). Means, standard errors and p-values obtained with a two-sided Student's t-test are shown.

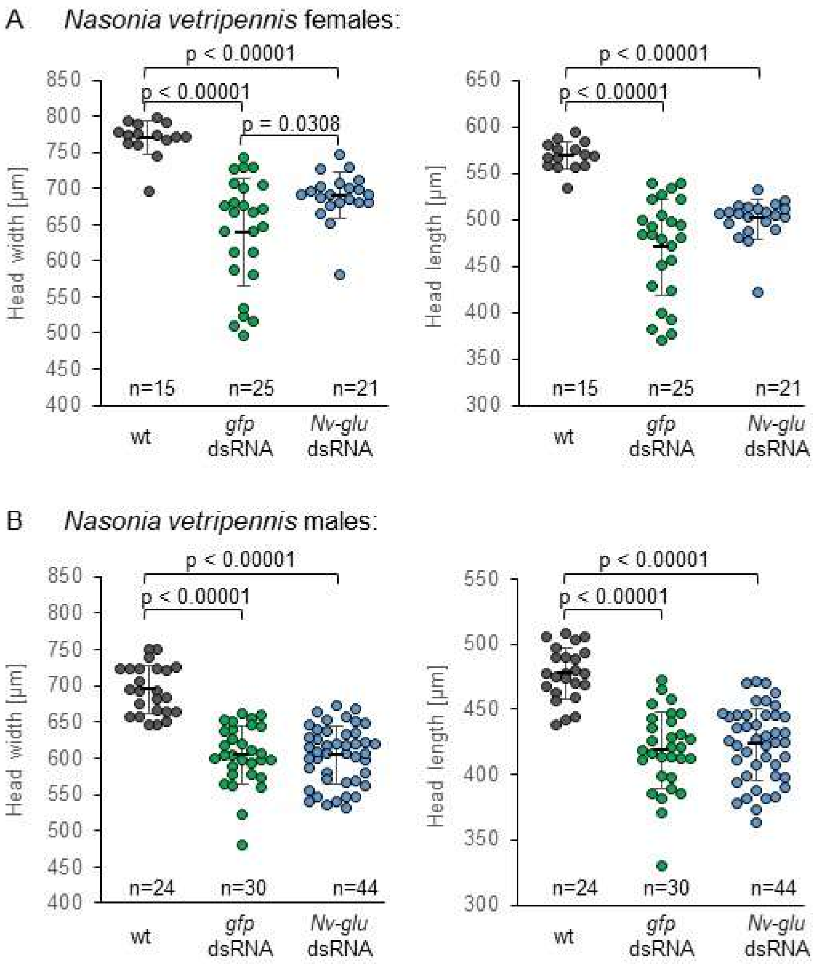

Supplementary Figure 9: The effect of larval injection on *N. vitripennis* adult head width and length. (A) *N. vitripennis* females and (B) *N. vitripennis* males left untreated (wt) or treated with *gfp* dsRNA or *Nv-glu* dsRNA. Means, standard deviations and p-values obtained with a two-tailed Mann-Whitney U test are shown.

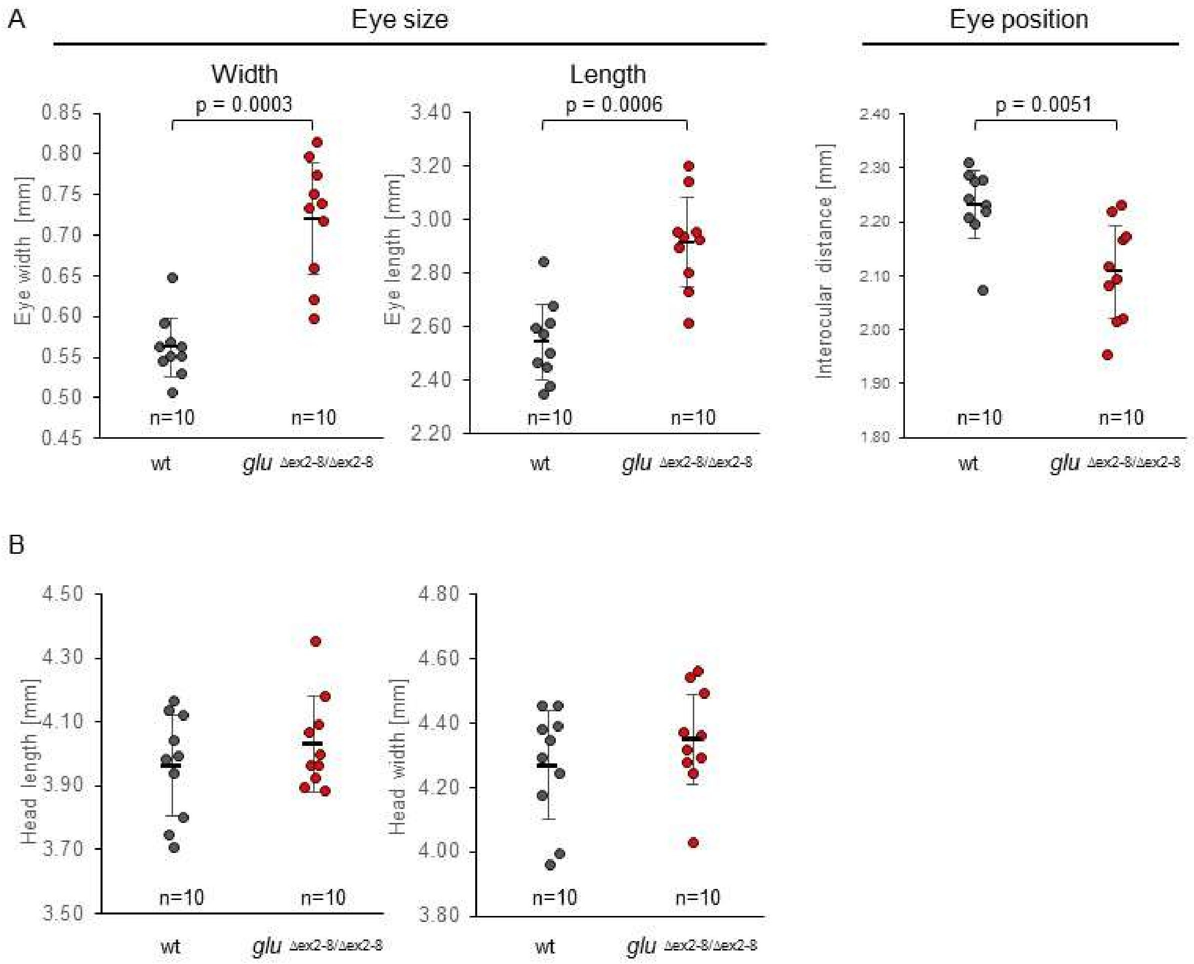

Supplementary Figure 10: Eye and head sizes in female mutants of the honeybee. (A) Eye width, eye length and eye position in *glu* $^{\Delta ex2\text{-}8/\Delta ex2\text{-}8}$ mutants and wild type (wt) honeybee females. (B) Head length and width in *glu* $^{\Delta ex2\text{-}8/\Delta ex2\text{-}8}$ mutants. Means, standard deviations and p-values (two-tailed Mann-Whitney U test) are shown.

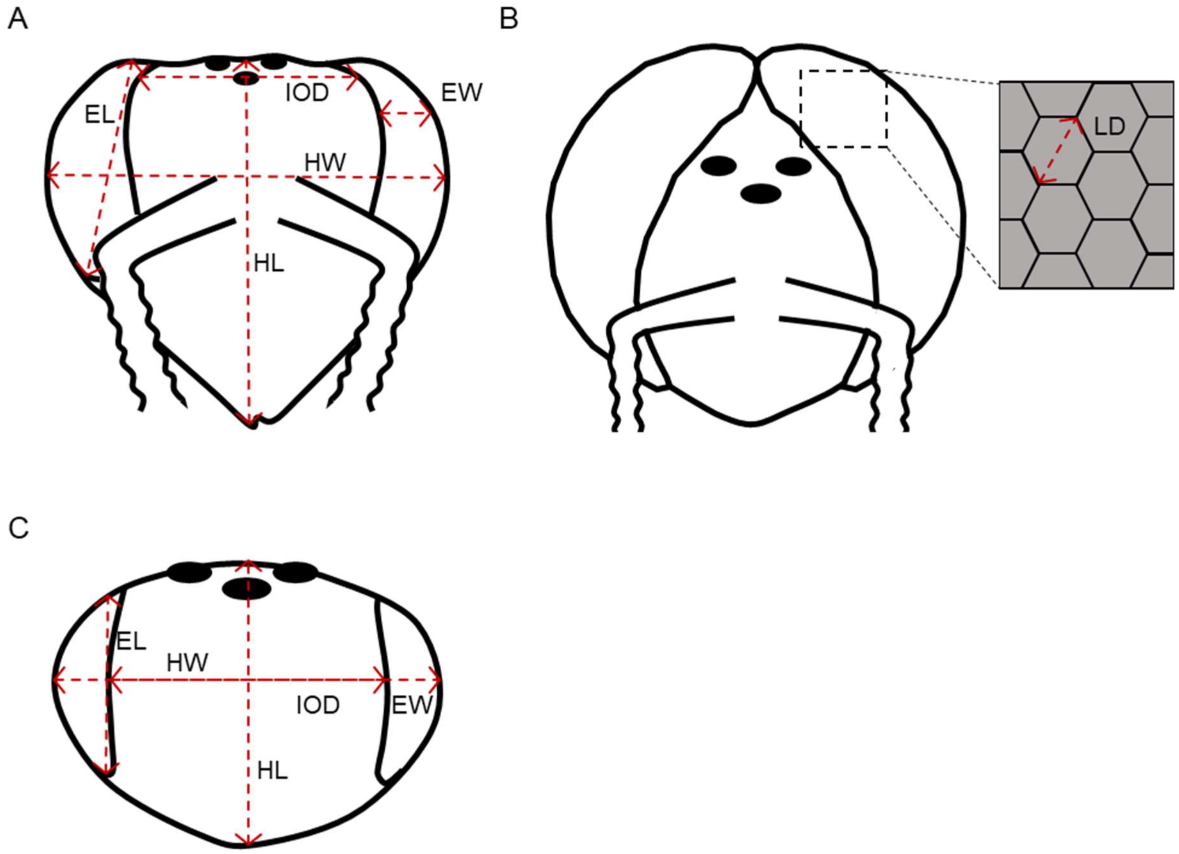

Supplementary Figure 11: The head and eye parameters of honeybee females (A), males (B) and the jewel wasp (C). EL: eye length; EW: eye width; IOD: interocular distance; HW: head width; HL: head length; LD: lens facet diameter.

Supplementary Table 1: Genotype sequences of *fem* $^{-/-}$ individuals. Wild type sequences are provided as reference. Target sites of *fem* sgRNA 2 are underlined.

Individual	sequence at target site aligned to wild type (wt) sequence
# 27	wt AGAGGACCAGAAG-------------GTACTCAAGTTAGT<br><br>sequence 1 ....-----------------............<br>sequence 2 ....--------------..............<br>sequence 3 .............GTACTCAGAGAAG..............<br>sequence 4 ....---------TTAG..............
# 36	wt AGAGGACCAGAAGGTACTCAAGTTAGT<br><br>allele 1/2 ....-..............
# 37	wt AGAGGACCAGAAG-GTACTCAAGTTAGT<br><br>allele 1/2 ....TC.............
# 44	wt AGAGGACCAGAAGGTACTCAAGTTAGT<br><br>allele 1 ....-..............<br>allele 2 ....--..............
# 56	wt AGAGGACCAGAAGGTACTCAAGTTAGT<br><br>allele 1/2 ....----..........
# 51	wt AGAGGACCAGAA-------------GGTACTCAAGTTAGT<br><br>allele 1 ............CTCAAGTAGGACC...............<br>allele 2 ....GTTATTTAGA-----..............
# 52	wt AGAGGACCAGAAGGTACTCAAGTTAGT<br><br>allele 1/2 ....-..............
# 53	wt AGAGGACCAGAAGGTACTCAAGTTAGT<br><br>allele 1/2 ....----...........
# 55	wt AGAGGACCAGAA-------------GGTACTCAAGTTAGT<br><br>allele 1 ....--------------..............<br>allele 2 ............GGTACTCAGAGAAG..............
# 58	wt AGAGGACCAGAAGGTACTCAAGTTAGT<br><br>allele 1/2 ....----...........
# 89	wt AGAGGACCAGAA----GGTACTCAAGTTAGT<br><br>allele 1/2 ....AGTT...............

Supplementary Table 2: Splicing of *dsx* transcripts in *fem* $^{-/-}$ individuals. The sex-specific transcripts of *doublesex* (*dsx*^{M} and *dsx*^{F}) were amplified in *fem* $^{-/-}$ first stage larvae and in wild type (wt) male and female controls.

	Individuals screened	Individuals with *dsx*^{F} splicing	Individuals with *dsx*^{M} splicing
Females *fem* $^{-/-}$	6	0	6 (100%)
wt male	6	0	6 (100%)
wt female	6	6 (100%)	0

Supplementary References

Eck, R.V., and Dayhoff, M.O. (1966). Atlas of protein sequence and structure, V. 3-5 (National Biomedical Research Foundation).

Moreau, C.S., Bell, C.D., Vila, R., Archibald, S.B., and Pierce, N.E. (2006). Phylogeny of the Ants: diversification in the age of Angiosperms. Science *312*, 101-4.

Munro, J.B., Heraty, J.M., Burks, R.A., Hawks, D., Mottern, J., Cruaud, A., Rasplus, J.-Y., and Jansta, P. (2011). A molecular phylogeny of the Chalcidoidea (Hymenoptera). PLoS One *6*, e27023.

Peters, R.S., Krogmann, L., Mayer, C., Donath, A., Gunkel, S., Meusemann, K., Kozlov, A., Podsiadlowski, L., Petersen, M., Lanfear, R., *et al.* (2017). Evolutionary history of the Hymenoptera. Curr. Biol. *27*, 1013-8.

Roth, A., Vleurinck, C., Netschitailo, O., Bauer, V., Otte, M., Kaftanoglu, O., Page, R.E., and Beye, M. (2019). A genetic switch for worker nutrition-mediated traits in honeybees. PLoS Biol. *17*, e3000171.

Tamura, K., Stecher, G., Peterson, D., Filipski, A., and Kumar, S. (2013). MEGA6: molecular evolutionary genetics analysis version 6.0. Mol. Biol. Evol. *30*, 2725-9.
