# Supplementary material for: The function and evolution of a genetic switch controlling sexually dimorphic eye differentiation in honeybees
Source: Nat Commun. 2023 Jan 28;14:463. doi: 10.1038/s41467-023-36153-4 (PMC9884244; doi:10.1038/s41467-023-36153-4)
Supplement: Supplementary file 2 — Description of Additional Supplementary Files [file 41467_2023_36153_MOESM2_ESM.pdf]

Description of Additional Supplementary Files

File Name: Supplementary Data 1
Description: Genotype sequences of *glu* $^{\Delta ex2\text{-}8/\Delta ex2\text{-}8}$, *glu* $^{tmC2H2/ex8stop}$, *glu* $^{tmC2H2/tmC2H2}$ and *glu* $^{ex8stop/ex8stop}$ individuals and sequences of oligonucleotides, sgRNAs and dsDNAs used.
