# Supplementary material for: The function and evolution of a genetic switch controlling sexually dimorphic eye differentiation in honeybees
Source: Nat Commun. 2023 Jan 28;14:463. doi: 10.1038/s41467-023-36153-4 (PMC9884244; doi:10.1038/s41467-023-36153-4)
Supplement: Supplementary file 4 — Reporting Summary [file 41467_2023_36153_MOESM4_ESM.pdf]

Corresponding author(s): Netschitailo, O., Beye, M.

Last updated by author(s): 2022-12-19

Reporting Summary

Nature Portfolio wishes to improve the reproducibility of the work that we publish. This form provides structure for consistency and transparency in reporting. For further information on Nature Portfolio policies, see our Editorial Policies and the Editorial Policy Checklist.

Please do not complete any field with "not applicable" or n/a. Refer to the help text for what text to use if an item is not relevant to your study.
For final submission: please carefully check your responses for accuracy; you will not be able to make changes later.

Statistics

For all statistical analyses, confirm that the following items are present in the figure legend, table legend, main text, or Methods section.

n/a	Confirmed	
☐	☒	The exact sample size (*n*) for each experimental group/condition, given as a discrete number and unit of measurement
☐	☒	A statement on whether measurements were taken from distinct samples or whether the same sample was measured repeatedly
☐	☒	The statistical test(s) used AND whether they are one- or two-sided<br>*Only common tests should be described solely by name; describe more complex techniques in the Methods section.*
☐	☒	A description of all covariates tested
☐	☒	A description of any assumptions or corrections, such as tests of normality and adjustment for multiple comparisons
☐	☒	A full description of the statistical parameters including central tendency (e.g. means) or other basic estimates (e.g. regression coefficient) AND variation (e.g. standard deviation) or associated estimates of uncertainty (e.g. confidence intervals)
☐	☒	For null hypothesis testing, the test statistic (e.g. *F*, *t*, *r*) with confidence intervals, effect sizes, degrees of freedom and *P* value noted<br>*Give P values as exact values whenever suitable.*
☒	☐	For Bayesian analysis, information on the choice of priors and Markov chain Monte Carlo settings
☒	☐	For hierarchical and complex designs, identification of the appropriate level for tests and full reporting of outcomes
☒	☐	Estimates of effect sizes (e.g. Cohen's *d*, Pearson's *r*), indicating how they were calculated

Our web collection on statistics for biologists contains articles on many of the points above.

Software and code

Policy information about availability of computer code

Data collection	uEye Cockpit (part of IDS Suite, 4.92, IDS; Germany) software was used for a UI-1240LE-C-HQ camera (IDS, Germany). The Dino-Lite Edge 5MP Digital Microscope (Dino-lire, Almere, The Netherlands) was used together with the DinoCapture 2.0 software. Measurement were taken with ImageJ 1.53q (National Institutes Health, USA).
Data analysis	LinRegPCR, 2017.1.0.0, HFRC was used for relative expression value calculations. CFX Manager 3.1 software (Bio–Rad, Hercules, USA) was used for RT-PCR curve analysis. The Galaxy online toolset (http://usegalaxy.com; Afgan et al., 2018) was used for amplicon sequence analysis. MEGA version 6 was used for sequence evolution analysis. The Benchling online toolset (Benchling [Biology Software]. (2019). Retrieved from https://benchling.com) was used for sgRNA design and off-target screening. Statistical analyses were performed using SigmaPlot 14.0 (Systat, San Jose, United States of America).

For manuscripts utilizing custom algorithms or software that are central to the research but not yet described in published literature, software must be made available to editors and reviewers. We strongly encourage code deposition in a community repository (e.g. GitHub). See the Nature Portfolio guidelines for submitting code & software for further information.

Data

Policy information about availability of data

All manuscripts must include a data availability statement. This statement should provide the following information, where applicable:
- Accession codes, unique identifiers, or web links for publicly available datasets
- A description of any restrictions on data availability
- For clinical datasets or third party data, please ensure that the statement adheres to our policy

The authors affirm that all data necessary for confirming the conclusions of the article are presented within the article, in the figures, supplementary information and data or in the source data file. Sex specific cds sequences of the glu gene were deposited in the data base NCBI under the accession codes OQ116780 and OQ 116781 (https://www.ncbi.nlm.nih.gov/gene). The databases PROSITE (https://prosite.expasy.org/; de Castro et al., 2006), InterPro (https://www.ebi.ac.uk/interpro/; Blum et al., 2021), Pfam (https://pfam.xfam.org; Mistry et al., 2020) and BLASTP tool of the NCBI database (https://blast.ncbi.nlm.nih.gov/Blast.cgi) used in this study are accessible online.

Human research participants

Policy information about studies involving human research participants and Sex and Gender in Research.

Reporting on sex and gender	No human research participants were involved in this study.
Population characteristics	*Describe the covariate-relevant population characteristics of the human research participants (e.g. age, genotypic information*
Recruitment	*Describe how participants were recruited. Outline any potential self-selection bias or other biases that may be present and*
Ethics oversight	*Identify the organization(s) that approved the study protocol.*

Note that full information on the approval of the study protocol must also be provided in the manuscript.

Field-specific reporting

Please select the one below that is the best fit for your research. If you are not sure, read the appropriate sections before making your selection.

☒ Life sciences ☐ Behavioural & social sciences ☐ Ecological, evolutionary & environmental sciences

Life sciences study design

All studies must disclose on these points even when the disclosure is negative.

Sample size	Sample size (usually around 10) was chosen on the basis of similar previously published studies of honeybee development. In Figure 5 only 5 individuals were obtained. Single data points are always presented in dot plots. Phenotype data were derived from single insects with independent induced mutations or knockdowns. They represent independent biological replicates.
Data exclusions	No data were excluded except for knockdown phenotype data of N. vitripennis, in which the injection procedure alone produced extreme outliers and variations for the size phenotypes of the head (Supplementary Figure 9). We used 1.5 times the standard deviation as criterion to remove such outliers (Birmingham et al., 2009) from head width or length data from both the control and treated group following the procedure (Figure 7d, Supplementary Figure 9).
Replication	All individual insects described in this study are the result of individual mutational or silencing events and thus represent independent biological replicates. No genetic identical lines were established. Embryos were obtained from different mothers with a diverse genetic background. Honeybee experiments were performed over the course of several weeks, with several injection sessions in which batches of control and mutated individuals were reared in parallel.
Randomization	Several batches of control and mutated bees were generated randomly in repeated experiments to exlude biases.
Blinding	Phenotyping was blinded: the investigator had no knowledge whether the insect belongs to the treatment/mutation or control group during phenotyping.

Reporting for specific materials, systems and methods

We require information from authors about some types of materials, experimental systems and methods used in many studies. Here, indicate whether each material, system or method listed is relevant to your study. If you are not sure if a list item applies to your research, read the appropriate section before selecting a response.

Materials & experimental systems

n/a	Involved in the study
☐	Antibodies
☐	Eukaryotic cell lines
☐	Palaeontology and archaeology
☒	Animals and other organisms
☐	Clinical data
☐	Dual use research of concern

Methods

n/a	Involved in the study
☐	ChIP-seq
☐	Flow cytometry
☐	MRI-based neuroimaging

Animals and other research organisms

Policy information about studies involving animals; ARRIVE guidelines recommended for reporting animal research, and Sex and Gender in Research

Laboratory animals	Nasonia vitripennis: strain AsymCx, cured of Wolbachia, adult males and females were used for crossing, RT-PCR (Figure 7a) and phenotype analysis (Figure 7d). Injection was performed into 2nd instar larvae, RT-PCR (Figure 7b) was performed on samples collected 3,4,5 days after injection.<br>Cimex lectularius: wild type, purchased from Insect Services GmbH (Berlin, Germany), male and female adults were used for analysis (Figure 7a).<br>Drosophila melanogaster: strain w1118, adult males and females (Figure 7a).<br>Tribolium castaneum: wild type, adult males and females (Figure 7a).<br>Apis mellifera carnica: wild type, naturally mated adult queens kept in an apiary were used for the production of female embryos for injections. injected females and males were reared in vitro until 4 pupal stage/eclosion. Phenotyping was performed on adults (Figure 5e) or pupae (4th pupal stage; Figure 5a-c, 6). RT PCR was performed on samples from embryos (FIgure 4a), L1 larvae (Figure 3a) or adults (Figure 3b, 4b).
Wild animals	Animals were not collected in the field but from eather lab cultures (D.melanogaster, T.castaneum, C.lectularius, N.vitripennis) or apiaries (A.mellifera) located in a research facility.
Reporting on sex	The findings apply to both sexes as mentioned in the result section/figures of this study. Adult animals were sexed according to their phenotype, sex of embryos/larva were eather checked by amplification of sex specific transcripts (fem/dsx transcripts) or study animals were derived from special crossings that yielded offspring of a cetrain sex: CO2 treated, non-mated honeybee queens or virgin N.vitripennis females produced male offspring.
Field-collected samples	Animals were not collected in the field but from eather lab cultures (Drosophila, Tribolium, Cimex, Nasonia) or apiaries.
Ethics oversight	The study was performed using insects that do not require ethical oversight in the countries this study was performed in (Germany, Netherlands).

Note that full information on the approval of the study protocol must also be provided in the manuscript.

This checklist template is licensed under a Creative Commons Attribution 4.0 International License, which permits use, sharing, adaptation, distribution and reproduction in any medium or format, as long as you give appropriate credit to the original author(s) and the source, provide a link to the Creative Commons license, and indicate if changes were made. The images or other third party material in this article are included in the article's Creative Commons license, unless indicated otherwise in a credit line to the material. If material is not included in the article's Creative Commons license and your intended use is not permitted by statutory regulation or exceeds the permitted use, you will need to obtain permission directly from the copyright holder. To view a copy of this license, visit http://creativecommons.org/licenses/by/4.0/
