# Supplementary material for: The function and evolution of a genetic switch controlling sexually dimorphic eye differentiation in honeybees
Source: Nat Commun. 2023 Jan 28;14:463. doi: 10.1038/s41467-023-36153-4 (PMC9884244; doi:10.1038/s41467-023-36153-4)
Supplement: Supplementary file 5 — Source Data [file 41467_2023_36153_MOESM5_ESM.zip › Source Data.pdf]

Figure 3a:

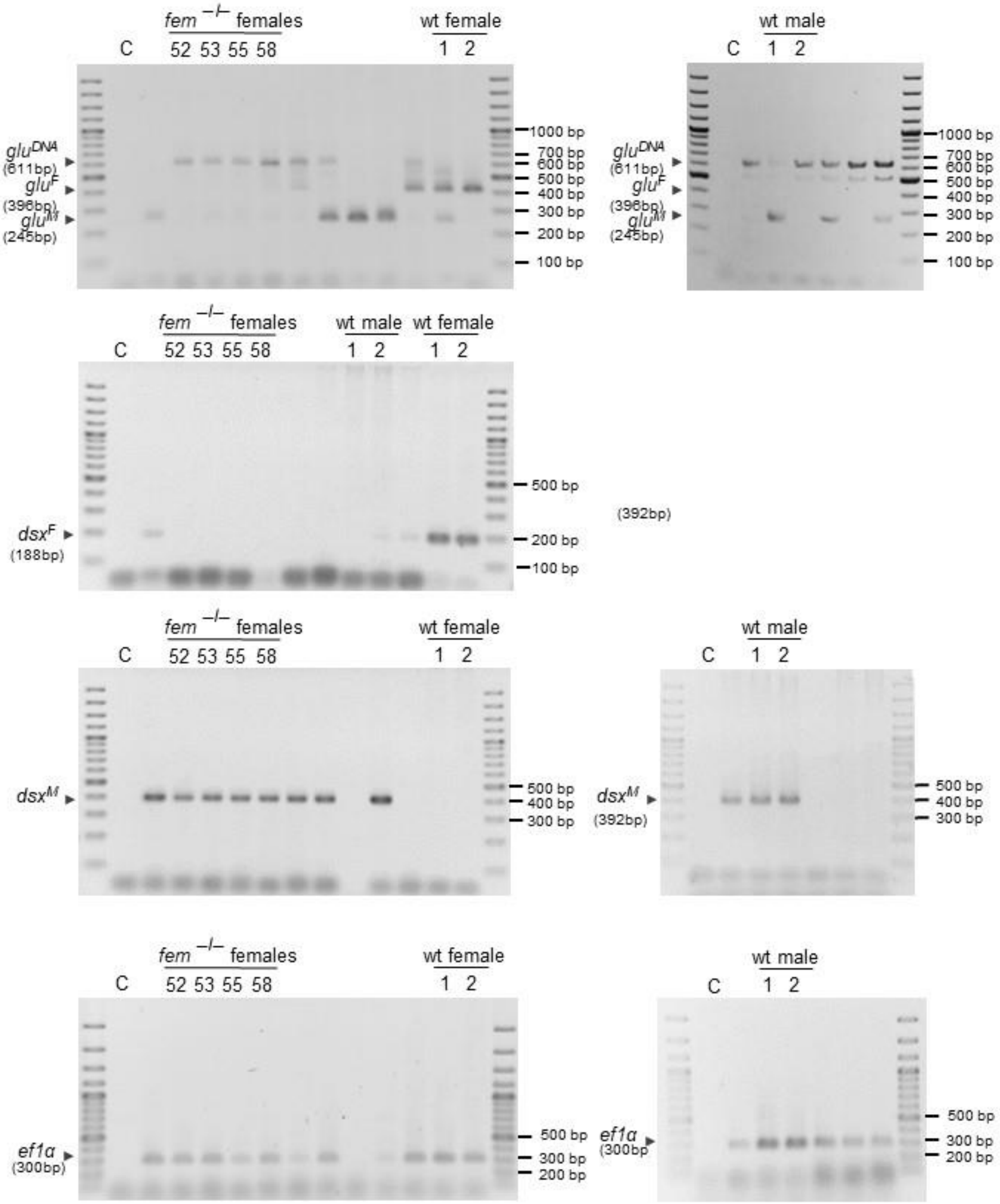
fem −/− females
52 53 55 58
wt female
1 2
C
wt male
1 2
glu^{DNA}
(611bp)
glu^{F}
(396bp)
glu^{M}
(245bp)
1000 bp
700 bp
600 bp
500 bp
400 bp
300 bp
200 bp
100 bp
dsx^{F}
(188bp)
(392bp)
dsx^{M}
(392bp)
ef1α
(300bp)

Figure 3b:

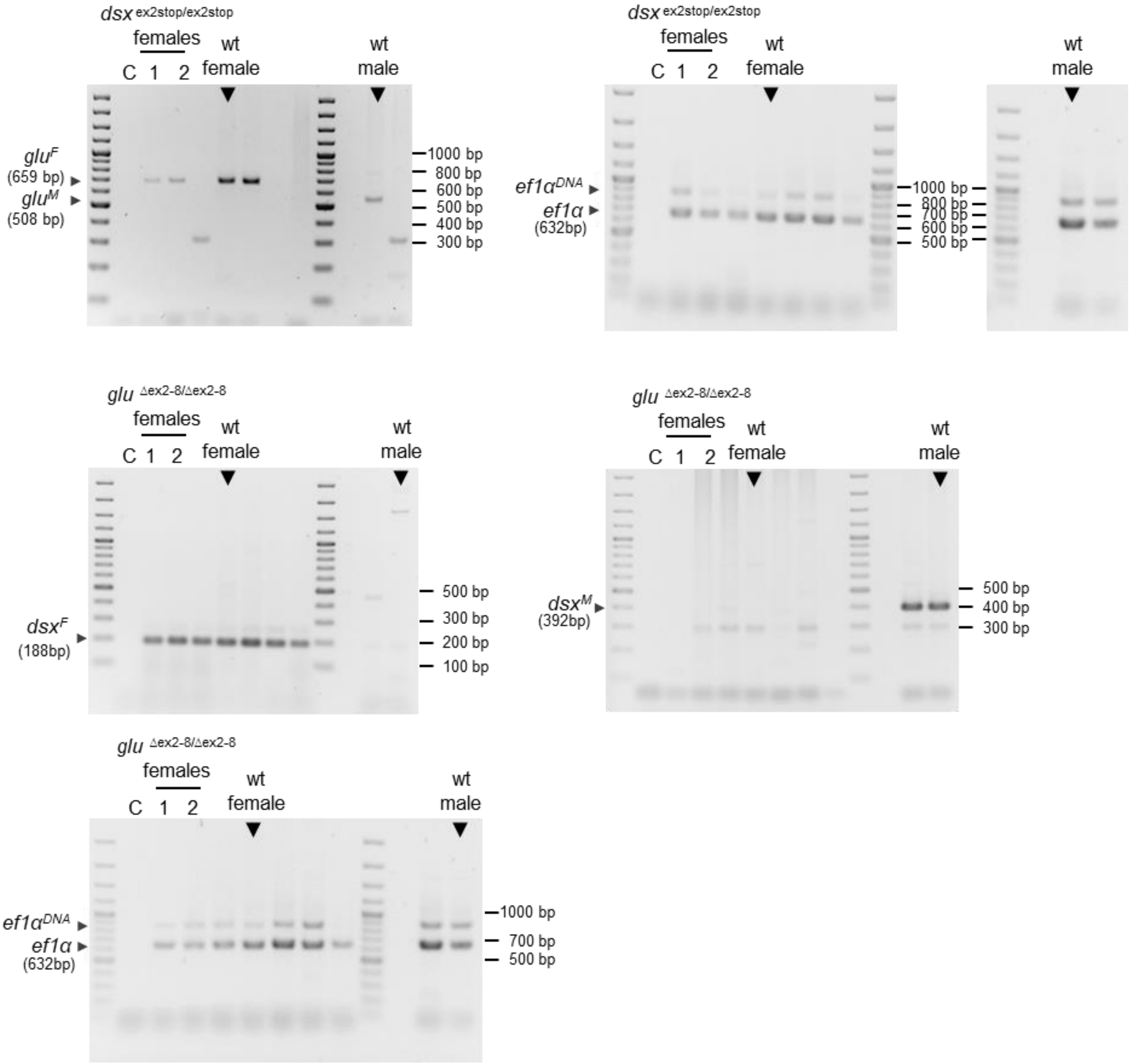

dsx ex2stop/ex2stop
females
C 1 2
wt female
wt male
gluF
(659 bp)
gluM
(508 bp)
1000 bp
800 bp
600 bp
500 bp
400 bp
300 bp
dsx ex2stop/ex2stop
females
C 1 2
wt female
wt male
ef1αDNA
ef1α
(632bp)
1000 bp
800 bp
700 bp
600 bp
500 bp
glu Δex2-8/Δex2-8
females
C 1 2
wt female
wt male
dsxF
(188bp)
500 bp
300 bp
200 bp
100 bp
glu Δex2-8/Δex2-8
females
C 1 2
wt female
wt male
dsxM
(392bp)
500 bp
400 bp
300 bp
glu Δex2-8/Δex2-8
females
C 1 2
wt female
wt male
ef1αDNA
ef1α
(632bp)
1000 bp
700 bp
500 bp

Figure 4a:

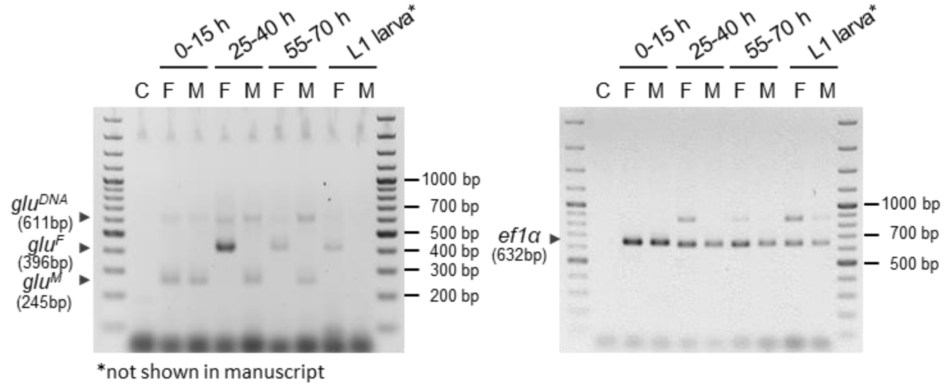

Figure 4b (and Supplementary Figure 3a-b):

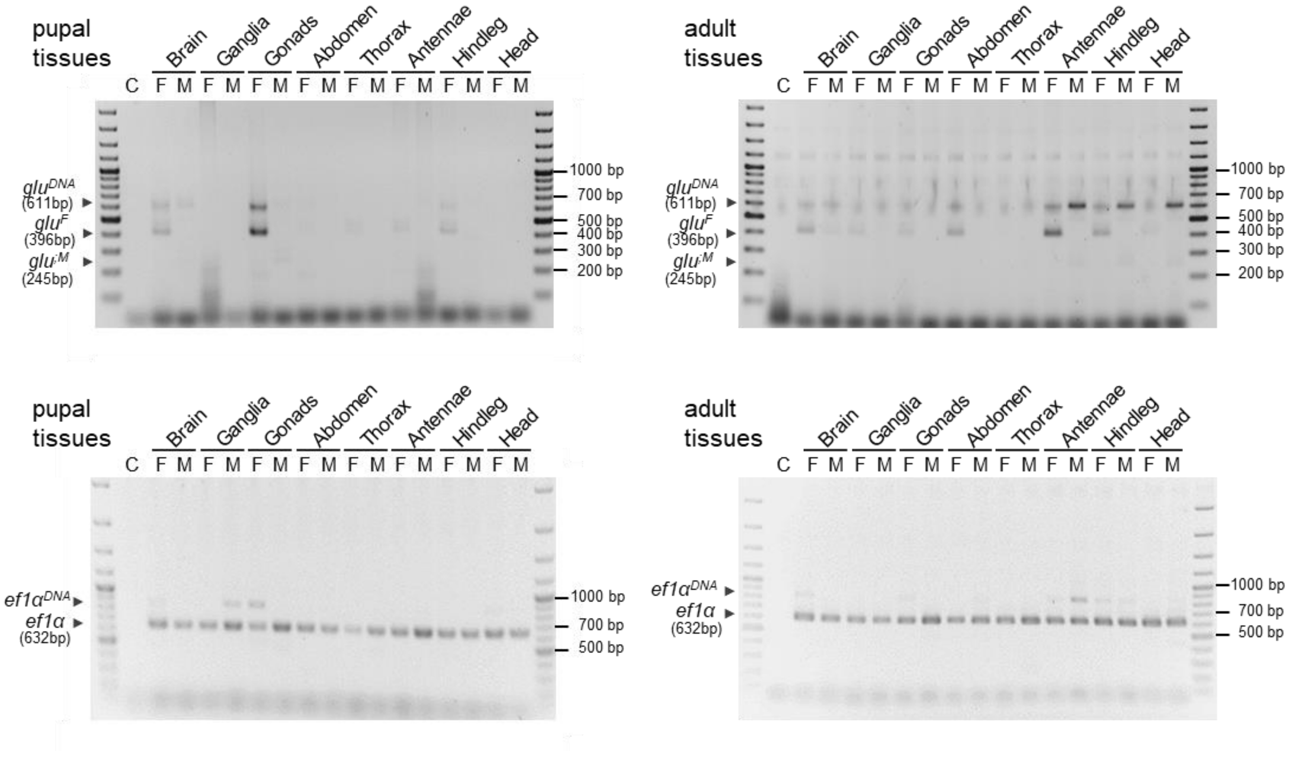

Figure 7a:

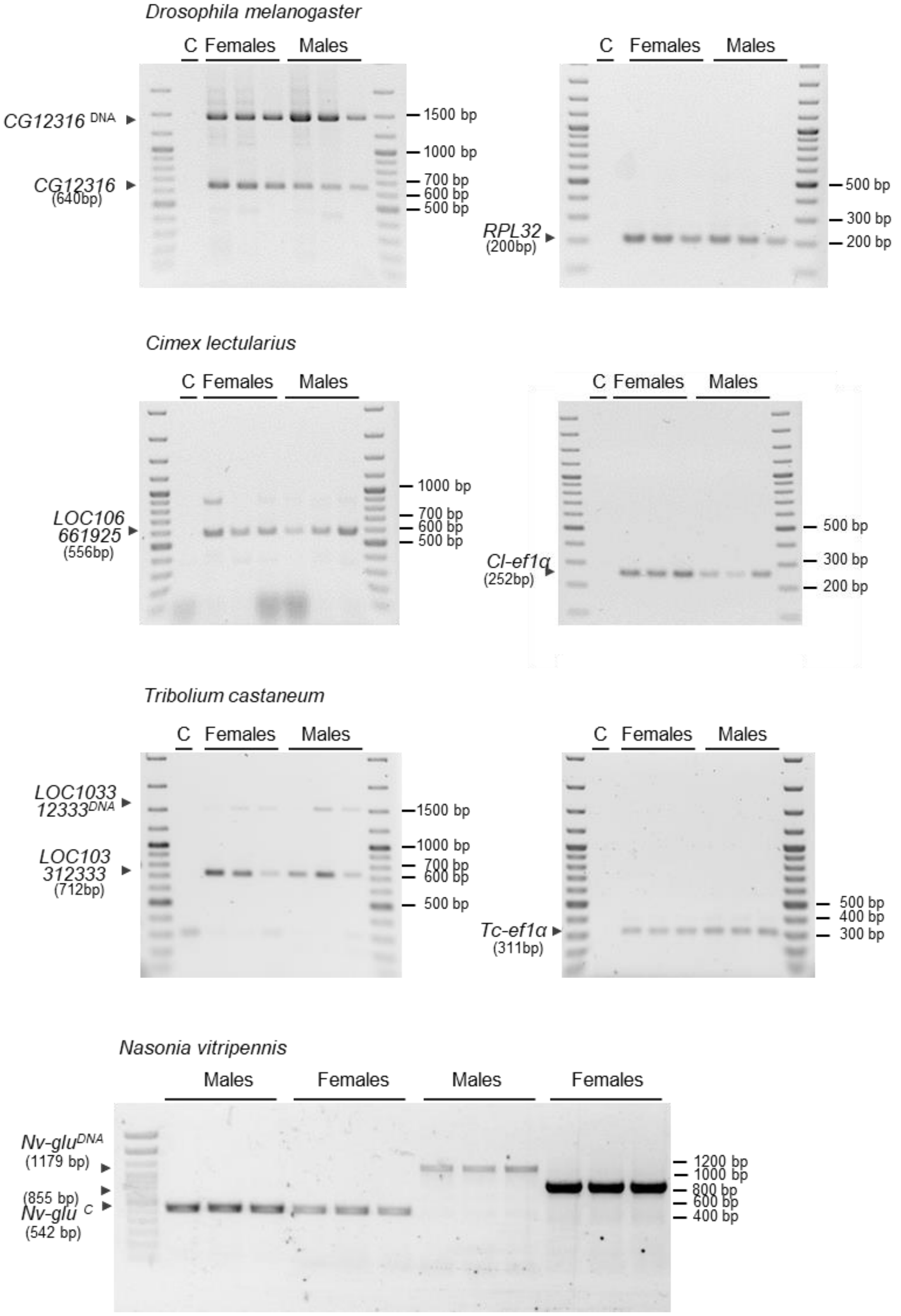

Drosophila melanogaster
C Females Males
CG12316 DNA
CG12316
(640bp)
1500 bp
1000 bp
700 bp
600 bp
500 bp
C Females Males
RPL32
(200bp)
500 bp
300 bp
200 bp
Cimex lectularius
C Females Males
LOC106
661925
(556bp)
1000 bp
700 bp
600 bp
500 bp
C Females Males
Cl-ef1α
(252bp)
500 bp
300 bp
200 bp
Tribolium castaneum
C Females Males
LOC1033
12333DNA
LOC103
312333
(712bp)
1500 bp
1000 bp
700 bp
600 bp
500 bp
C Females Males
Tc-ef1α
(311bp)
500 bp
400 bp
300 bp
Nasonia vitripennis
Males Females Males Females
Nv-gluDNA
(1179 bp)
(855 bp)
Nv-glu C
(542 bp)
1200 bp
1000 bp
800 bp
600 bp
400 bp

Figure 7b:

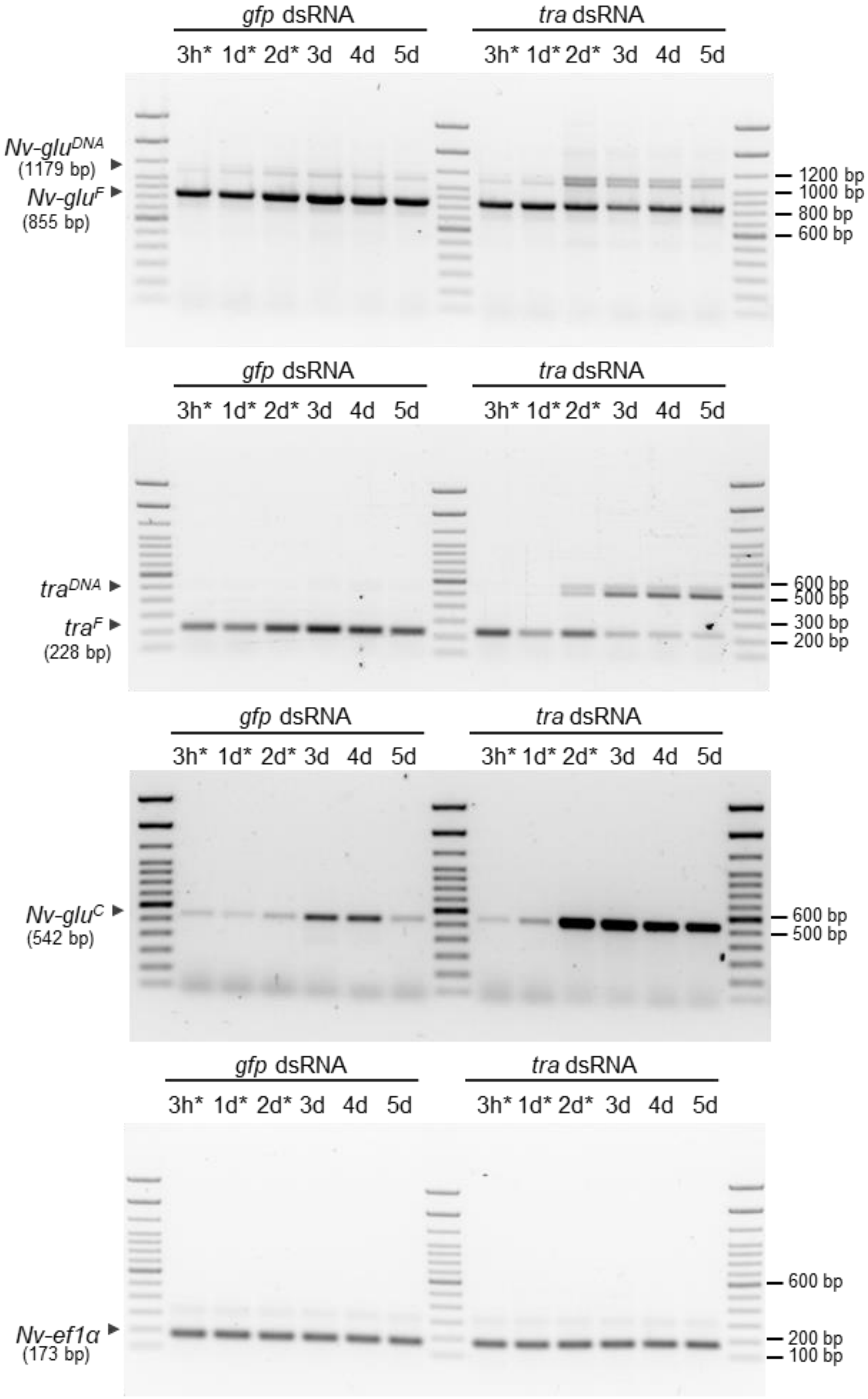

*data not shown in the paper. Samples collected 2 hours to two days after dsDNA treatment
